# Lyn restrains lupus via kinase-independent mechanisms that limit Toll-like receptor activation and type I interferon responsiveness

**DOI:** 10.1126/sciadv.adz1726

**Published:** 2025-10-17

**Authors:** Elan L’Estrange-Stranieri, Timothy A. Gottschalk, Anne M. Kong, Mhairi J. Maxwell, Ee Shan Pang, Evelyn Tsantikos, David M. Tarlinton, Meredith O’Keeffe, Mark D. Wright, Margaret L. Hibbs

**Affiliations:** ^1^ Department of Immunology, School of Translational Medicine, Monash University, Melbourne, VIC 3004, Australia.; ^2^ Department of Biochemistry and Molecular Biology, Biomedicine Discovery Institute, Monash University, Clayton, VIC 3800, Australia.

## Abstract

Lyn phosphorylates inhibitory immunoreceptors to terminate signaling; consequently, Lyn deficiency in mice causes hyperactive immune cells and lupus-like autoimmune disease. Lyn may also suppress autoimmunity independent of its kinase activity through inhibitory protein-protein binding interactions, although the importance of this mechanism is unclear. To analyze the kinase-independent functions of Lyn, mice expressing a catalytically inactive mutant of Lyn were generated and their phenotype compared to Lyn-deficient mice. Disease progression was blunted in Lyn kinase-dead mice indicating a contribution for kinase-independent Lyn functions in restraining autoantibody production, glomerulonephritis, Toll-like receptor signaling, and splenomegaly. Further comparative analyses identified an exclusive role for the kinase-dependent functions of Lyn in regulating B cell receptor signaling, dendritic cell phenotype, and type I interferon production. By contrast, interferon-stimulated gene expression and the regulation of thymic epithelial cell development and T cell selection are previously unidentified, exclusively kinase-independent functions for Lyn. Collectively, these findings further our understanding of the nuanced roles of Lyn in health and disease.

## Teaser

Lyn restrains lupus by kinase-independent mechanisms that curb Toll-like receptor signaling and interferon responsiveness.

## INTRODUCTION

Systemic lupus erythematosus (SLE; lupus) is a chronic autoinflammatory disease characterized by the production of antinuclear antibodies (ANAs) and type I interferon (IFN-I), with severe presentations affecting the kidney, resulting in lupus nephritis (LN) (*1*). Lyn^−/−^ mice are a well-studied model of SLE, exhibiting hyperactive B lymphocytes and myeloid cells that drive autoantibody production and systemic inflammation (*2*–*4*) culminating in immune complex (IC)–mediated glomerulonephritis (*5*–*8*). Lyn is a nonreceptor protein tyrosine kinase that is expressed broadly and is critical to B lymphocyte and myeloid cell regulation by phosphorylating a network of inhibitory immunoreceptors and downstream phosphatases. Lyn deficiency is therefore thought to result in SLE-like disease due to a loss of Lyn-driven inhibitory target phosphorylation (*9*). Supporting this, Lyn-deficient mice exhibit abrogated phosphorylation of inhibitory molecules, including FcγRIIb, PIR-B, SIRPα, CD22, SHP1, and SHIP1 (*10*–*13*). Furthermore, the deficiency of these targets similarly results in SLE-like disease in mice (*14*–*18*).

Protein kinases are known to have important functions independent of their catalytic activity. Kinase-independent functions include protein-protein interactions where kinases act as scaffolding proteins, allosteric regulators, or competitively bind to targets and block their interactions with other targets (*19*). Studies of a kinase-dead Lyn mutant (LynKD; p.K275X) have also demonstrated Lyn to have kinase-independent functions in vitro. An early report showed that B cell antigen receptor (BCR) hyperresponsiveness of Lyn-deficient avian B cells (DT40 cell line) was abrogated by the expression of kinase-dead Lyn (*20*). Another study reported Lyn binding to p53 in the nucleus upon DNA damage, independent of kinase activity, to promote p53-mediated gene expression in epithelial cell lines (*21*). Using human embryonic kidney (HEK) 293T cells, kinase-dead Lyn was further shown to competitively bind the transcription factor interferon regulatory factor (IRF) 5 downstream of MyD88 and Toll-like receptor (TLR) 7/9 stimulation, blocking access to IRF5 from IKKβ and TRAF6, thereby inhibiting IRF5 activation (*22*). The authors postulated that the loss of this kinase-independent inhibitory function of Lyn, particularly in dendritic cells (DCs), may be critical to the SLE-like disease of Lyn-deficient mice, by promoting aberrant IRF5-driven IFN-I production and proinflammatory cytokine-induced inflammation (*22*). This is in keeping with previous studies that have shown that MyD88-dependent signaling is essential to disease of Lyn-deficient mice (*2*, *3*, *23*, *24*) and DCs as being similarly central to disease given the severe autoimmune pathology of CD11c-Cre x Lyn^flox/flox^ mice (*3*). Yet, other in vitro investigations have refuted this kinase-independent inhibitory function of Lyn in the TLR-IRF pathway, instead showing that Lyn inhibits the IRF family downstream of TLRs by phosphorylating IRFs to direct their ubiquitination and proteasomal degradation (*25*). Therefore, there is contention over the legitimacy of Lyn’s kinase-independent functions. Furthermore, although Lyn-deficient DCs are assumed to be key drivers of disease (*3*, *22*, *26*), a detailed assessment of the DC compartment in Lyn-deficient mice has not previously been reported. This is warranted considering that the CD11c-Cre x Lyn^flox/flox^ mice (*3*, *26*) do not represent a model of DC-specific Lyn deficiency as CD11c is expressed broadly in hematopoietic cells, including in natural killer cells, eosinophils, neutrophils, monocytes, and macrophages (*27*). Mice harboring *N*-ethyl-*N*-nitrosourea mutations in *Lyn* that affect its kinase activity have been described. WeeB mice harbor an E260G mutation in the glycine-rich loop of the N-terminal lobe of the kinase domain (*28*), whereas Mld4 mice exhibit a T410K mutation in the highly conserved Src activation loop (*29*). Both mutants exhibited BCR hyperresponsiveness, and whereas WeeB mice were reported to develop late onset autoimmune disease (*28*), autoimmune pathology was attenuated in Mld4 mice (*29*), suggesting that Lyn has the ability to suppress autoimmune disease via kinase-independent functions. Nonetheless, the mutations in these strains are not bona fide kinase-dead mutations, being situated outside of the adenosine triphosphate (ATP)–binding site.

Elucidating the cell types, pathways, and mechanisms by which Lyn prevents SLE-like disease is important in furthering our understanding of how SLE develops in humans and in enabling these inhibitory mechanisms to be targeted for SLE therapies. To address this and, in particular, to investigate the kinase-independent functions of Lyn in regulating autoimmune pathology, mice expressing a kinase-dead mutant of Lyn were generated and phenotypically compared to Lyn-deficient mice. We reasoned that pathways altered in both strains would signify those regulated by Lyn’s kinase activity, whereas those normalized in kinase-dead Lyn mutant mice would identify those controlled by Lyn’s kinase-independent functions. We demonstrate that kinase-independent functions of Lyn contribute substantially to the restraint of autoimmunity, and by our comparative analyses of immune phenotyping and disease progression, we have begun to define kinase-dependent and independent functions of Lyn, which may ultimately have the potential to uncover therapeutic targets for the treatment of lupus.

## RESULTS

### Mice expressing kinase-dead Lyn have attenuated autoimmune pathology compared to Lyn-deficient mice

**Fig. 1.**
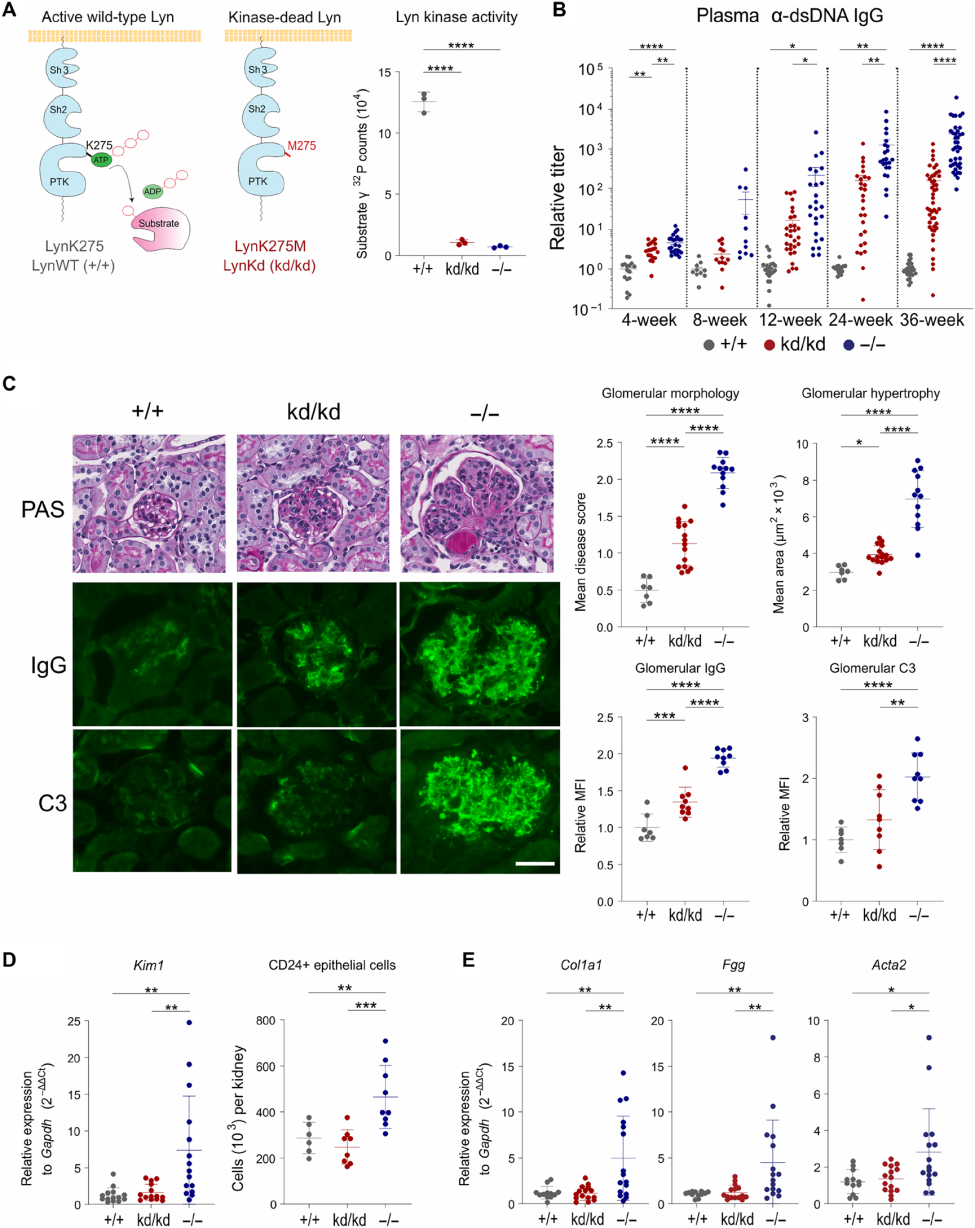
Mice expressing kinase-dead Lyn have attenuated autoimmune pathology compared to Lyn-deficient mice. (**A**) Left: Schematic of the Lyn structure and function highlighting the point mutation in LynKD (K275M) that renders LynKD catalytically inactive, in contrast to active WT Lyn. Right: Tyrosine kinase activity of Lyn immunoprecipitated from BMDMs of the indicated mice (*n* = 3 per genotype) in the presence of SOP and [γ-^32^P]ATP. Results representative of two independent experiments. (**B**) Anti-dsDNA IgG plasma titers from bleeds of mice at the indicated ages, determined by ELISA, samples normalized to Lyn^+/+^ controls within each aging cohort (*n* = 10 to 28 Lyn^+/+^, 13 to 47 Lyn^kd/kd^, and 11 to 39 Lyn^−/−^ mice per time point). (**C**) Left: Representative images of cortical glomeruli from 36-week-old mice; kidney sections stained with PAS (top), α-IgG-AF488 (middle), or α-C3-FITC (bottom). Scale bar, 50 μm. Right top: Quantification of the glomerular pathology of PAS-stained kidney sections, mean of *n* ≥ 50 cortical glomeruli per mouse (*n* = 7 Lyn^+/+^, 16 Lyn^kd/kd^, and 12 Lyn^−/−^ mice). Bottom: Quantification of IgG and C3 glomerular MFI relative to Lyn^+/+^ mice (relative MFI), *n* = 7 Lyn^+/+^, 9 Lyn^kd/kd^, and 9 Lyn^−/−^ mice. (**D**) Left: *Kim1* (*Havcr1*) expression in kidney tissue determined by RT-PCR from 36-week-old (*n* = 7 Lyn^+/+^, 9 Lyn^kd/kd^, and 9 Lyn^−/−^) mice. Right: CD24+ epithelial cells enumerated by flow cytometry of perfused kidneys from 24-week-old (*n* = 6 Lyn^+/+^, 8 Lyn^kd/kd^, and 9 Lyn^−/−^) mice, data compiled from two independent experiments. (**E**) Profibrotic gene expression in kidney tissue from 36-week-old (*n* = 12 Lyn^+/+^, 15 Lyn^kd/kd^, and 16 Lyn^−/−^) mice. Horizontal lines on graphs indicate means ± SD [(A), (C), (D), and (E)] or SEM (B). *P < 0.05, **P < 0.01, ***P < 0.001, and ****P < 0.0001 by one-way ANOVA with Holm-Šídák’s multiple comparisons test.

Mice expressing catalytically inactive Lyn, herein referred to as kinase-dead Lyn (LynKD, Lyn^kd/kd^), were generated on the C57BL/6 genetic background using targeted homologous recombination to introduce a K275M point mutation, removing the ATP-binding site in the Lyn kinase domain (Fig. 1A and fig. S1A). Expression of the LynKD mutant was confirmed in whole-cell lysates (WCLs) of cultured bone marrow–derived macrophages (BMDMs) from Lyn^kd/kd^ mice, although it was moderately reduced compared to Lyn expression in C57BL/6 mice (fig. S1, B and C). The absence of kinase activity was confirmed in an in vitro kinase assay using immunoprecipitated Lyn (Fig. 1A).

An assessment of the autoimmune disease profile of Lyn^kd/kd^ mice was undertaken relative to Lyn-deficient (Lyn^−/−^) mice, with matched C57BL/6 mice [wild type (WT); Lyn^+/+^] used as a healthy comparator. Immune profiling was undertaken before disease onset and up to 36 weeks of age when inflammatory pathology is established in Lyn^−/−^ mice with class-switched autoantibodies against nuclear antigens and severe IC-mediated glomerulonephritis (*30*).

In Lyn^−/−^ mice, titers of anti– double-stranded DNA (dsDNA) immunoglobulin G (IgG) were detectable as early as 4 weeks of age and increased exponentially over the course of disease development, being maximal in 36-week-old mice (Fig. 1B). Lyn^kd/kd^ mice also had detectable anti-dsDNA IgG; however, titers were substantially reduced compared to Lyn-deficient mice (Fig. 1B).

Lyn^kd/kd^ mice were found to have greatly reduced kidney pathology at 36 weeks, showing only mild glomerular hypertrophy and disrupted morphology compared to the advanced disease seen in Lyn^−/−^ mice, with glomeruli characterized by severe lobularity, segmental necrosis and advanced hypertrophy (Fig. 1C and fig. S1D). Furthermore, Lyn^kd/kd^ mice exhibited mild glomerular IgG deposition and no complement C3 fixation, in contrast to the extensive IgG and C3 deposits seen in Lyn^−/−^ glomeruli (Fig. 1C). Distinct from the Lyn^−/−^ kidney, gene expression of Kidney-Injury Molecule-1 (*Kim1/Havcr1*), a biomarker of active LN (*31*, *32*), was not induced in Lyn^kd/kd^ kidney and neither were CD24+ renal progenitor cells that typically proliferate during kidney damage (*33*, *34*) (Fig. 1D and fig. S1E). Similarly, Lyn^kd/kd^ kidney showed no enhancement of profibrotic gene expression including type I collagen (*Col1a1*), fibrinogen (*Fgg*), and smooth muscle actin (*Acta2*), unlike the Lyn^−/−^ kidney (Fig. 1E). No major sex differences were observed in disease parameters of Lyn^kd/kd^ mice (fig. S2, A to F); however, kidney histopathology was worse in female Lyn^−/−^ mice (fig. S2B) whereas all other disease features were equally distributed amongst males and females.

To assess whether autoimmune disease in Lyn^kd/kd^ mice could be exacerbated by greater pathogen burden from the environment, a cohort of mice was aged in a low-barrier facility. Disease was promoted in Lyn^−/−^ mice under low-barrier conditions as severe disease manifested earlier at 30 weeks of age, whereas Lyn^kd/kd^ mice exhibited only mild renal disease and inflammation (fig. S3, A to C) like mice aged under specific pathogen–free conditions. These analyses suggest that Lyn^kd/kd^ mice have robust attenuation of autoimmune IC-mediated nephritis compared to complete Lyn deficiency, demonstrating that Lyn has important kinase-independent inhibitory functions in suppressing autoimmune pathology in vivo.

### Renal inflammation is limited by kinase-dead Lyn

**Fig. 2.**
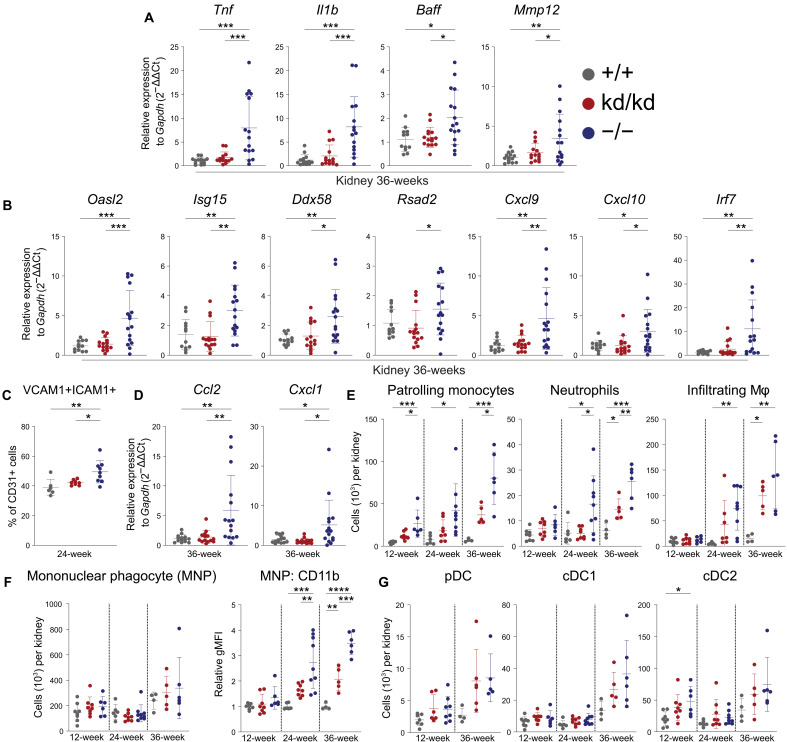
Renal inflammation is limited in Lyn^kd/kd^ mice. (**A**) Proinflammatory gene expression in kidney tissue determined by RT-PCR from 36-week-old mice (*n* = 14 Lyn^+/+^, 14 Lyn^kd/kd^, and 15 Lyn^−/−^) and (**B**) ISG expression (*n* = 12 Lyn^+/+^, 15 Lyn^kd/kd^, and 16 Lyn^−/−^ mice). (**C**) Endothelial cell activation determined by flow cytometry of perfused kidney from 24-week-old (*n* = 6 Lyn^+/+^, 8 Lyn^kd/kd^, and 9 Lyn^−/−^) mice, data compiled from two independent experiments. (**D**) Kidney gene expression of myeloid chemoattractants determined by RT-PCR from 36-week-old (*n* = 14 Lyn^+/+^, 14 Lyn^kd/kd^, and 15 Lyn^−/−^) mice. (**E** to **G**) Renal myeloid populations enumerated by flow cytometry of perfused kidneys at the indicated ages (*n* = 4 to 8 Lyn^+/+^, 5 to 8 Lyn^kd/kd^, and 6 to 9 Lyn^−/−^ mice per time point), data compiled from at least four independent experiments. Relative gMFI normalized to Lyn^+/+^ mice. Horizontal bars indicate means ± SD. *P < 0.05, **P < 0.01, ***P < 0.001, and ****P < 0.0001 by one-way ANOVA with Holm-Šídák’s multiple comparisons test.

Glomerular IC deposition precipitates an inflammatory insult on the kidney, and therefore, the nature of renal inflammation was investigated. Kidneys from 36-week-old Lyn^−/−^ mice showed elevated proinflammatory gene expression (*Tnf*, *Il1b*, *Baff*, and *Mmp12*) and type I IFN-stimulated gene (ISG) expression, in contrast to Lyn^kd/kd^ mice whose kidney expression profile was comparable to WT mice (Fig. 2, A and B).

Consistent with this, renal endothelial cell activation in Lyn^kd/kd^ mice was similar to WT mice (Fig. 2C), as was the kidney expression of genes encoding monocyte and neutrophil chemoattractants CCL2 and CXCL1, respectively (Fig. 2D), whereas these were enhanced in Lyn^−/−^ mice. This corresponded to a reduction in infiltrating patrolling monocytes and neutrophils in Lyn^kd/kd^ kidneys, although kidney-infiltrating macrophages were similarly expanded in both Lyn^kd/kd^ and Lyn^−/−^ mice at 36 weeks (Fig. 2E and fig. S4A), whereas numbers of classical and intermediate monocytes were generally unchanged compared to WT mice (fig. S4B).

Renal mononuclear phagocytes (MNPs) are the most abundant kidney immune cell population, being embryonically derived and sharing features of both macrophages and DCs (*35*–*37*). Although numbers of MNPs were unchanged, a marked up-regulation of CD11b was seen on Lyn^−/−^ MNPs, which did not occur to the same degree on Lyn^kd/kd^ MNPs, consistent with reduced activation of this population (Fig. 2F and fig. S4A).

Conventional DC type 1 (cDC1), conventional DC type 2 (cDC2), and plasmacytoid dendritic cells (pDCs) were relatively rare in the kidney, and numbers were generally unchanged in Lyn^−/−^ and Lyn^kd/kd^ mice compared to control mice (Fig. 2G and fig. S4A). Infiltrating lymphocytes were also investigated; however, no differences in natural killer cells, T lymphocytes, or B lymphocytes were seen between Lyn^−/−^ and Lyn^kd/kd^ mice, except that expansion of γδ T cells was only seen in the Lyn^−/−^ kidney (fig. S5, A and B). The contribution of T cells to Lyn^−/−^ renal disease in situ was, however, unclear as no increase in effector T cell gene expression (*Granzyme B, Ifng*, and *Il17*) was found in Lyn^−/−^ kidneys (fig. S5C).

Together, these findings show that kinase-dead Lyn protects against immune cell infiltration into kidney and in situ renal inflammation that is observed in Lyn^−/−^ mice.

### Kinase-dead Lyn suppresses myeloid cell responses to TLR agonists and myeloid-driven inflammation in vivo

**Fig. 3.**
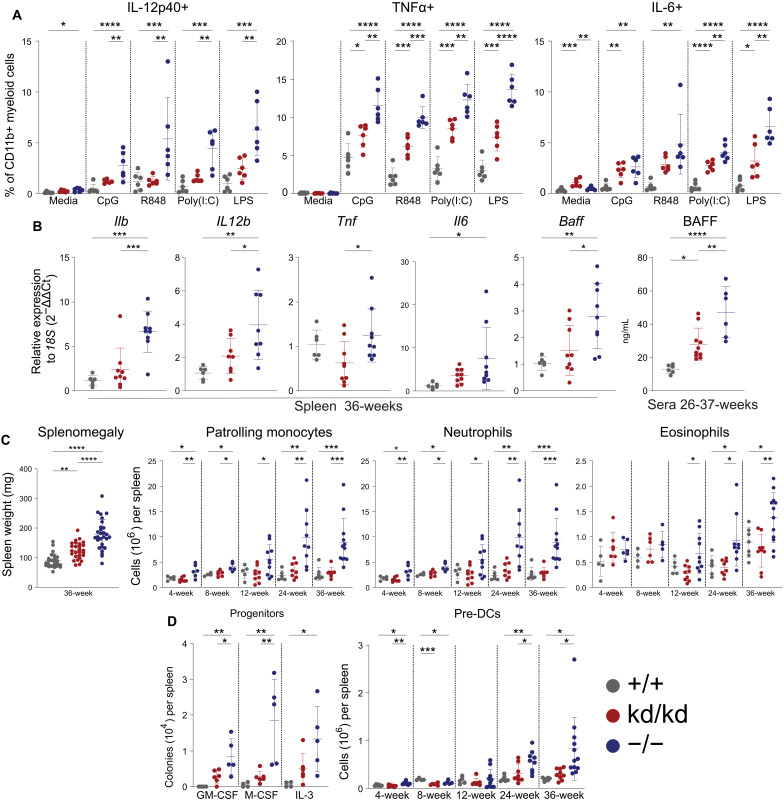
Kinase-dead Lyn suppresses myeloid-driven inflammation in vivo. (**A**) Splenocytes stimulated ex vivo for 4 hours with 1 μM CpG-C ODN 2395, R848 (5 μg/ml), Poly(I:C) (5 μg/ml), LPS (5 μg/ml), or media in the presence of GolgiPlug and intracellular cytokine staining assessed by flow cytometry (*n* = 6 per genotype; 4- to 6-week-old mice), data representative of two independent experiments. (**B**) Spleen gene expression determined by RT-PCR from 36-week-old (*n* = 6 Lyn^+/+^, 9 Lyn^kd/kd^, and 9 Lyn^−/−^) mice and sera concentration of BAFF determined by ELISA from 26- to 27-week-old (*n* = 6 Lyn^+/+^, 10 Lyn^kd/kd^, and 6 Lyn^−/−^) mice. (**C**) Left: Spleen weights from 36-week-old (*n* = 24 Lyn^+/+^, 27 Lyn^kd/kd^, and 28 Lyn^−/−^) mice. Right: Myeloid spleen cell subsets enumerated by flow cytometry at the indicated ages. (**D**) Left: Splenocytes from 26- to 30-week-old (*n* = 4 Lyn^+/+^, 6 Lyn^kd/kd^, and 5 Lyn^−/−^) mice stimulated with M-CSF (10 ng/ml), IL-3 (10 ng/ml), or GM-CSF (10 ng/ml) and colonies counted after 7 days, data compiled from two independent experiments. Right: Splenic pre-DC progenitors (defined as B220−SiglecH−F4/80−CD64−MHCII−CD11c+; fig. S12A) enumerated by flow cytometry at the indicated ages. (C and D) Flow cytometry data compiled from eight independent experiments (*n* = 5 to 7 Lyn^+/+^, 6 to 9 Lyn^kd/kd^, and 5 to 12 Lyn^−/−^ mice per time point). Horizontal bars indicate means ± SD. *P < 0.05, **P < 0.01, ***P < 0.001, and ****P < 0.0001 by one-way ANOVA with Holm-Šídák’s multiple comparisons test.

To investigate the mechanism by which kinase-dead Lyn limits inflammation, myeloid cell responses to TLR agonists were assessed. CD11b+ splenocytes (which predominantly consists of monocytes and granulocytes; fig. S6A) from Lyn^−/−^ mice exhibited a greater propensity to produce interleukin-12p40 (IL-12p40), tumor necrosis factor–α (TNFα), and IL-6 in response to ex vivo stimulation with CpG-C, R848, polyinosinic:polycytidylic acid [Poly(I:C)], and lipopolysaccharide (LPS), compared to WT cells, with Lyn^kd/kd^ cells showing an intermediate phenotype (Fig. 3A and fig. S6B). Consistent with this, gene expression of proinflammatory cytokines in the spleen of aged Lyn^kd/kd^ mice was equivalent to the WT spleen, and circulating levels of BAFF were reduced compared to Lyn^−/−^ mice (Fig. 3B). Furthermore, aged Lyn^kd/kd^ mice showed restrained splenomegaly (Fig. 3C), no myeloid cell expansion (Fig. 3C and fig. S7A), and a small but not statistically significant induction of splenic progenitor cells (which underpins the splenic hematopoiesis phenotype) (Fig. 3D), indicating attenuated inflammation.

Splenic macrophages were found to be largely unchanged in number in Lyn^−/−^ and Lyn^kd/kd^ mice compared to controls (fig. S7B). TLR-induced cytokine production from splenic macrophages of Lyn^−/−^ mice was variable, in keeping with previous findings (*26*, *38*), and similar to Lyn^kd/kd^ macrophage responses (fig. S8, A and B).

Overall, the data suggest that the expression of kinase-dead Lyn is sufficient to suppress the propagated inflammatory and myeloproliferative hallmarks of Lyn^−/−^ mice, in part by restraining responsiveness to TLR-agonism in monocytes and granulocytes.

### Lyn^kd/kd^ B cells are hyperresponsive to BCR stimulation but show inhibited TLR activation

**Fig. 4.**
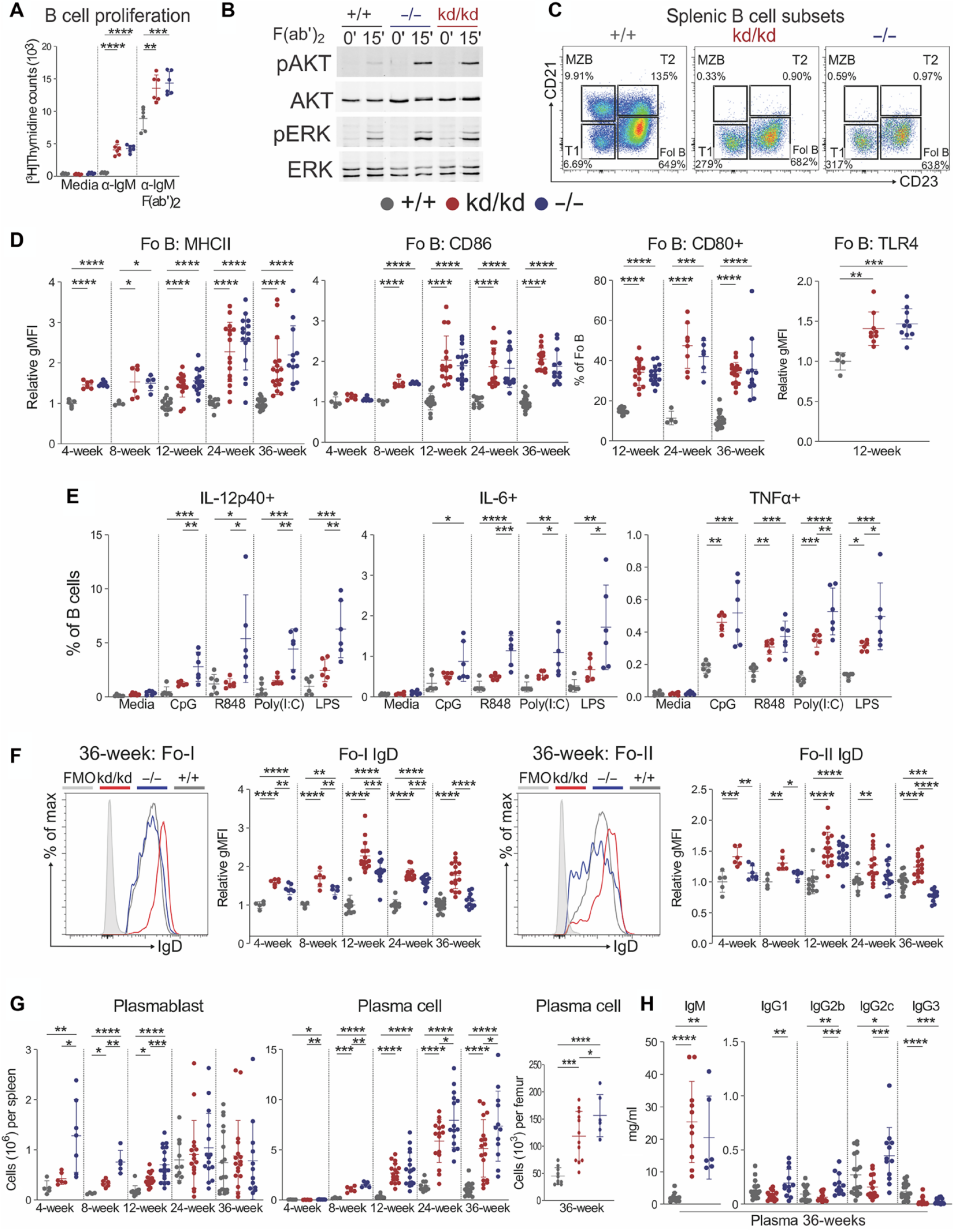
LynKD B cells show enhanced BCR signaling but inhibited TLR pathway activation. (**A**) Splenic B cell proliferation assessed by [^3^H]thymidine incorporation following 72-hour stimulation with anti-IgM (10 μg/ml), F(ab′)_2_ anti-IgM (10 μg/ml), or media (*n* = 6 per genotype; 8- to 12-week-old mice), data representative of two independent experiments. (**B**) Immunoblot of phospho-AKT, phospho-ERK1/2, AKT, and ERK1/2 from purified splenic B cells unstimulated or stimulated for 15 min with F(ab′)_2_ anti-IgM (20 μg/ml). Pooled densitometry data shown in fig. S9A. Data representative of three independent experiments; uncropped Western blots shown in fig. S9 [(B) to (D)]. (**C**) Representative flow cytometry plot of splenic B lymphocytes (CD19+B220+CD138−CD11c−) from 36-week-old mice. (**D**) Fo B cell activation marker expression, relative gMFI normalized to mean of Lyn^+/+^ mice. (**E**) Splenocytes stimulated for 4 hours with R848 (5 μg/ml), Poly(I:C) (5 μg/ml), 1 μM CpG-C ODN 2395, LPS (5 μg/ml), or media in the presence of GolgiPlug and intracellular cytokine staining of B cells assessed by flow cytometry (*n* = 6 per genotype; 4- to 6-week-old mice), data representative of two independent experiments. (**F**) Representative histograms of IgD expression on Fo-I and Fo-II B cells and relative gMFI from mice at the indicated ages. FMO, fluorescence minus one. (**G**) Left: Plasmablast splenocyte numbers. Right: Plasma cell numbers in the spleen and BM of mice at the indicated ages. (**H**) Immunoglobulin plasma concentration determined by ELISA from 36-week-old (*n* = 10 to 18 Lyn^+/+^, 11 to 16 Lyn^kd/kd^, and 6 to 18 Lyn^−/−^) mice. (D, F, and G) Data compiled from 11 independent experiments (*n* = 4 to 18 Lyn^+/+^, 6 to 17 Lyn^kd/kd^, and 5 to 18 Lyn^−/−^ mice per time point). Horizontal bars indicate means ± SD. *P < 0.05, **P < 0.01, ***P < 0.001, and ****P < 0.0001 by one-way ANOVA with Holm-Šídák’s multiple comparisons test.

B cells are necessary for the autoimmune pathology of Lyn-deficient mice (*39*), with Lyn inhibiting BCR signaling by phosphorylating inhibitory co-receptors (*2*). Consistent with a loss of this inhibitory function, Lyn^kd/kd^ splenic B cells were hyperactive to BCR cross-linking, on par with Lyn^−/−^ B cells, demonstrating increased proliferation and activation signaling (Fig. 4, A and B, and fig. S9, A to D). Augmented BCR signaling strength in Lyn^−/−^ mice results in mature B cell lymphopenia due to enhanced negative selection of B cells at the transitional type 2 (T2) and T3 stages of development (*40*, *41*). Similarly, Lyn^kd/kd^ mice exhibited an almost complete absence of T2 and marginal zone B (MZB) cells, as well as markedly depleted mature splenic follicular (Fo) B cells, lymph node B cells, and circulating and recirculating B cells, whereas numbers of early B cell developmental populations (pre/pro-B cells, immature B cells, and splenic T1 B cells) were less affected (Fig. 4C and fig. S10, A to C). Furthermore, Lyn^kd/kd^ and Lyn^−/−^ Fo B cells displayed an activated phenotype, with up-regulated costimulatory marker expression and increased TLR4 (Fig. 4D and fig. S11A). Age-associated B cells (ABCs) were also investigated and found to be expanded as a proportion of total B cells but unchanged in number in Lyn^−/−^ and Lyn^kd/kd^ spleens compared to controls (figs. S10B and S11B). Thus, the shared perturbations in the B cell compartment indicate that BCR hyperactivity occurs to the same extent in vivo in Lyn^kd/kd^ mice as Lyn^−/−^ mice.

Nonetheless, when stimulated with TLR agonists, splenic B cells from 4- to 6-week-old Lyn^kd/kd^ mice had a lower propensity for IL-12p40 and IL-6 production compared to matched Lyn^−/−^ B cells. Similarly, whereas B cell TNFα induction was marginal overall, frequencies of TNFα+ Lyn^kd/kd^ B cells were also less than Lyn^−/−^ B cells in response to LPS or Poly(I:C) stimulation (Fig. 4E and fig. S11C). This suggests that, although kinase-dead Lyn does not restrain BCR signaling, it instead limits responsiveness within the TLR pathway in B cells. Lyn^kd/kd^ Fo-I and Fo-II B cell populations also exhibited marked up-regulation of IgD compared to WT and Lyn^−/−^ mice (Fig. 4F), a known tolerizing mechanism of anergic autoreactive B cells (*42*).

Antibody-secreting cell (ASC) differentiation was also restrained as Lyn^kd/kd^ plasmablast numbers were reduced in young mice, and plasmacytosis was moderated with age in the spleen and BM (Fig. 4G). IgM hyperglobulinemia occurred to the same extent; however, aged Lyn^kd/kd^ mice had lower titers of IgG1, IgG2b, and IgG2c isotypes compared to Lyn-deficient mice (Fig. 4H), which, coupled with attenuated IgG ANA titers in Lyn^kd/kd^ mice (Fig. 1B), is consistent with restrained class switching of autoreactive B cells (*24*).

Collectively, the results suggest that, despite exhibiting defective BCR signaling and an activated phenotype, akin to total loss of Lyn, kinase-dead Lyn suppresses the synergistic activation of the TLR pathway in B cells, which may promote an anergic phenotype and potentially suppresses autoreactive B cell class switching to pathogenic IgG isotypes.

### cDCs are similarly dysregulated in Lyn^−/−^ and Lyn^kd/kd^ mice

Lyn deficiency in the DC compartment has been suggested as a key driver of disease (*3*), with further reports proposing that Lyn inhibits DCs principally through kinase-independent mechanisms (*22*). To address this, a detailed assessment of the cDC compartment was undertaken in Lyn^−/−^ and Lyn^kd/kd^ mice.

Staining of splenocytes from aged Lyn^−/−^ mice revealed that most CD11c+MHCII+ cells were ABCs and macrophages (fig. S12A) and, if unaccounted for, contaminated the cDC2 gate (fig. S12B). Aged Lyn^−/−^ mice also exhibited an expanded CD11c+MHCII− subset that was SIPRα+CD11b−CD4−CD8α− (fig. S13, A and B), consistent with an intrasplenic pre-cDC progenitor population (*43*) that likely expanded with age due to extramedullary hematopoiesis (Fig. 3D). Thus, herein cDC1s were identified as B220−SiglecH−F4/80−CD64−CD11c+MHCII+CD8α+SIRPα−, cDC2s as B220−SiglecH−F4/80−CD64−CD11c+MHCII+CD8α−SIRPα+, and pre-DCs as B220−SiglecH−F4/80−CD64−CD11c+MHCII− (fig. S13A).

**Fig. 5.**
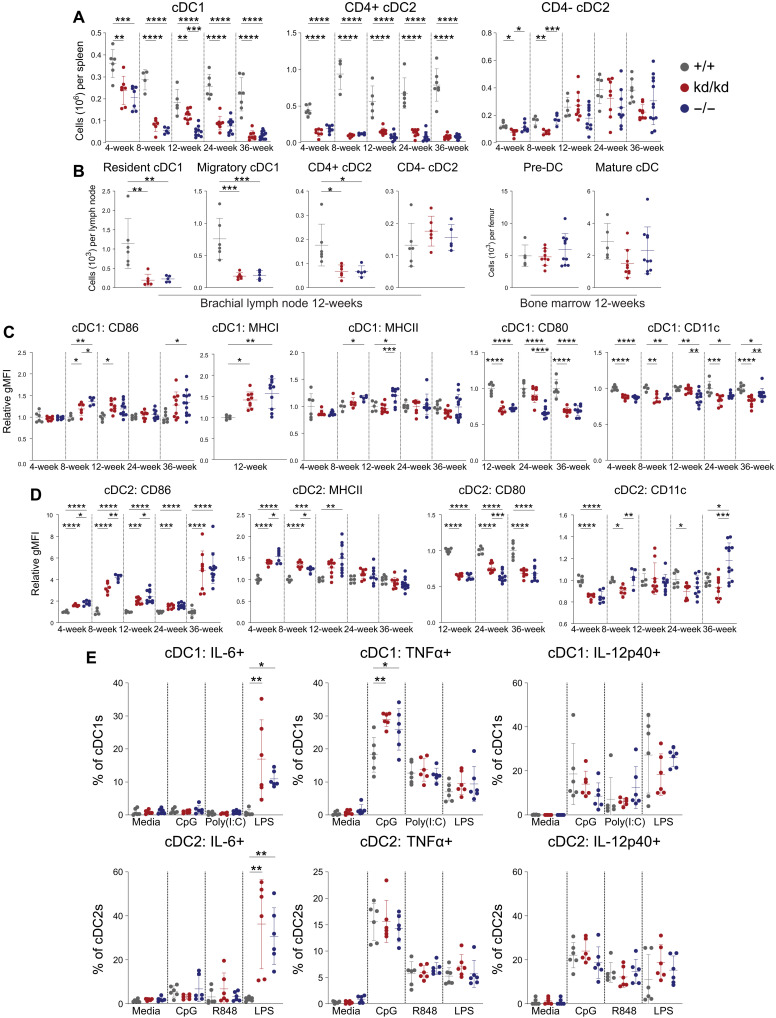
cDCs are similarly dysregulated in Lyn^−/−^ and Lyn^kd/kd^ mice. (**A**) Splenic cDC numbers enumerated by flow cytometry at the indicated ages. (**B**) Left: cDC numbers in brachial lymph node. Right: cDC numbers in BM from 12-week-old (*n* = 5 to 6 Lyn^+/+^, 6 to 9 Lyn^kd/kd^, and 5 to 10 Lyn^−/−^) mice, data compiled from three independent experiments. (**C**) Splenic cDC1 and (**D**) splenic cDC2 activation marker expression; relative gMFI normalized to Lyn^+/+^ mean. (**E**) Splenocytes stimulated for 4 hours in the presence of GolgiPlug plus 1 μM CpG-C ODN 2395, Poly(I:C) (5 μg/ml), R848 (5 μg/ml), LPS (5 μg/ml), or media and intracellular cytokine staining of cDC1s and cDC2s assessed by flow cytometry (*n* = 6 per genotype; 4- to 6-week-old mice), data representative of two independent experiments. [(A), (C), and (D)] Data compiled from eight independent experiments (*n* = 4 to 7 Lyn^+/+^, 6 to 9 Lyn^kd/kd^, and 5 to 12 Lyn^−/−^ mice per time point). Horizontal bars indicate means ± SD. *P < 0.05, **P < 0.01, ***P < 0.001, and ****P < 0.0001 by one-way ANOVA with Holm-Šídák’s multiple comparisons test.

Immunophenotyping of cDCs in Lyn^−/−^ and Lyn^kd/kd^ mice revealed a specific depletion of cDC1s and CD4+ cDC2s in secondary lymphoid tissue as early as 4 weeks of age, whereas numbers of CD4− cDC2s were generally unchanged (Fig. 5, A and B, and fig. S13, A and D). BM pre-cDC progenitors (Lineage−CD11c+MHCII−) (*44*) and cDC numbers were normal, suggesting that the reduction in peripheral cDC populations was not due to a gross developmental perturbation (Fig. 5B and fig. S13C).

cDC1s are efficient in the cross-presentation of antigen on major histocompatibility complex (MHC) class I (MHCI) to stimulate CD8+ T cells (*45*) and showed some signs of activation in Lyn^−/−^ and Lyn^kd/kd^ mice, including enhanced MHCI expression as well as CD86 and MHCII up-regulation at certain time points. However, other activation markers such as CD80 and CD11c were consistently down-regulated (Fig. 5C).

cDC2s are efficient CD4+ T cell stimulators (*45*), and consistent up-regulation of CD86 in Lyn^−/−^ and Lyn^kd/kd^ mice was observed; however, CD80 was reduced (Fig. 5D). cDC2 MHCII expression was elevated in young Lyn^−/−^ and Lyn^kd/kd^ mice but comparable to controls at aged time points, whereas CD11c was variable (Fig. 5D). Overall, the surface marker expression of cDCs from Lyn^−/−^ and Lyn^kd/kd^ mice is indicative of a similarly dysregulated phenotype.

Some signs of Lyn^−/−^ and Lyn^kd/kd^ cDC hyperactivity were evident when stimulated with TLR agonists, including increased propensity to produce IL-6 to LPS stimulation and TNFα to CpG-C stimulation; however, overall, Lyn^−/−^ and Lyn^kd/kd^ cDC responses were similar to WT cDCs (Fig. 5E and fig. S14, A and B).

Given the similar perturbations observed in the cDC compartment in Lyn^−/−^ and Lyn^kd/kd^ mice, the kinase activity of Lyn appears critical for maintaining normal cDC homeostasis. Furthermore, as no differences were found between Lyn^−/−^ and Lyn^kd/kd^ cDCs, the kinase-independent functions of Lyn do not appear to play a prominent inhibitory role in cDCs, indicating that the disease attenuation of Lyn^kd/kd^ mice is unlikely to be mediated by cDC inhibition.

### Kinase-dead Lyn does not inhibit pDC IFN-I production and instead restrains immune cell responsiveness to IFN-I

**Fig. 6.**
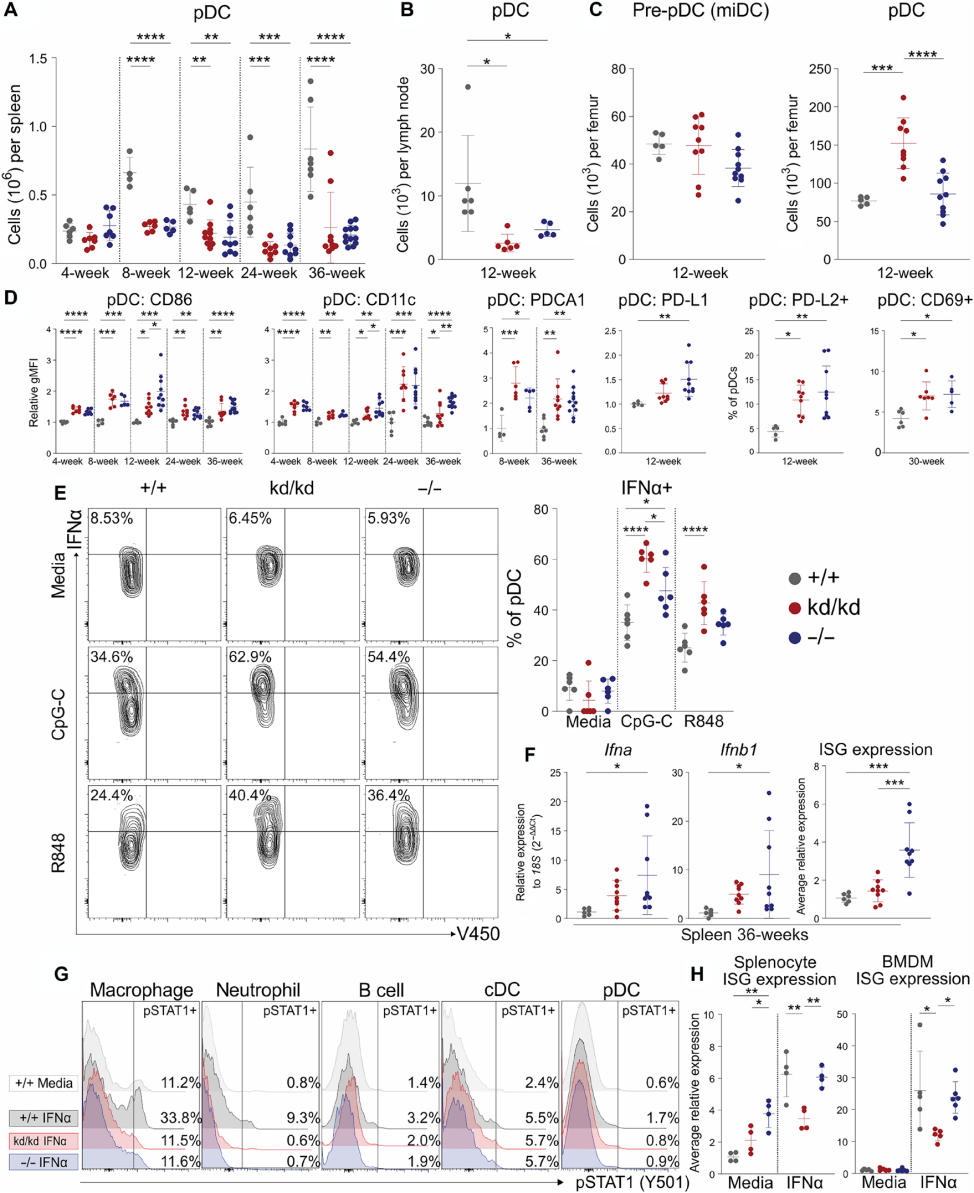
Kinase-dead Lyn does not inhibit pDC IFN-α production and instead suppresses ISG transcription. (**A**) Splenic pDCs and (**B**) brachial lymph node pDCs enumerated by flow cytometry at the indicated ages, data compiled from nine independent experiments (*n* = 4 to 7 Lyn^+/+^, 6 to 9 Lyn^kd/kd^, and 5 to 12 Lyn^−/−^ mice per time point). (**C**) BM pre-pDCs (Lineage-SiglecH+MHCII-B220low; fig. S13C) and mature pDCs enumerated by flow cytometry from 12-week-old (*n* = 5 Lyn^+/+^, 9 Lyn^kd/kd^, and 9 Lyn^−/−^) mice, data compiled from two independent experiments. (**D**) Splenic pDC activation marker expression; relative gMFI normalized to Lyn^+/+^ mean. Data compiled from nine independent experiments (*n* = 4 to 7 Lyn^+/+^, 6 to 9 Lyn^kd/kd^, and 5 to 12 Lyn^−/−^ mice per time point). (**E**) Left: Representative plots of splenic pDC intracellular IFN-α staining following 4-hour stimulation with 1 μM CpG-C ODN 2395, R848 (5 μg/ml), or media in the presence of GolgiPlug. Right: Quantification (*n* = 6 per genotype; 4- to 6-week-old mice). (**F**) Expression of *Ifna*, *Ifnb1*, and ISGs (average expression of seven ISGs shown in fig. S15A) in spleen tissue determined by RT-PCR from 36-week-old (*n* = 6 Lyn^+/+^, 9 Lyn^kd/kd^, and 9 Lyn^−/−^) mice. (**G**) Representative histograms of phospho-STAT1 assessed by flow cytometry of splenocytes cultured with media or IFN-α (1000 IU/ml) for 30 min (*n* = 5 per genotype; 6-week-old mice), quantification shown in fig. S15B. (**H**) ISG expression (average of six to seven ISGs shown in fig. S15, C and D) determined by RT-PCR from splenocytes and BMDM cultured with media or IFN-α (1000 IU/ml) for 8 hours (*n* = 4 to 5 Lyn^+/+^, 4 to 5 Lyn^kd/kd^, and 4 to 6 Lyn^−/−^ mice). Horizontal bars indicate means ± SD. *P < 0.05, **P < 0.01, ***P < 0.001, and ****P < 0.0001 by one-way ANOVA with Holm-Šídák’s multiple comparisons test.

The lupus-like disease of Lyn-deficient mice can be ameliorated by IFNAR1 deficiency (*46*), and therefore, the IFN-I response in Lyn kinase-dead mice was investigated. Immunophenotyping revealed that splenic and lymph node pDCs, the potent IFN-I–producing cells, were depleted equally in Lyn^−/−^ and Lyn^kd/kd^ mice compared to controls from 8 weeks of age onward (Fig. 6, A and B, and fig. S13A). BM pre-pDC progenitors (miDCs) (*47*, *48*) and mature pDCs were not reduced in number in either Lyn^−/−^ or Lyn^kd/kd^ mice, indicating no impairment in pDC development (Fig. 6C and fig. S13C).

Lyn^−/−^ and Lyn^kd/kd^ pDCs showed consistent signs of activation across all time points, with enhanced activation marker expression (CD86 and CD11c) and elevated IFN-I–induced marker expression (PDCA1, PD-L1, PD-L2, and CD69) (*49*) (Fig. 6D). Furthermore, stimulation of splenocytes from 4- to 6-week-old mice with TLR agonists induced IFN-α production, which was enhanced in Lyn^−/−^ pDCs, and further enhanced in Lyn^kd/kd^ pDCs compared to WT pDCs (Fig. 6E). Corroborating elevated IFN-α production, spleen gene expression of *Ifna* and *Ifnb1* was augmented in aged Lyn^−/−^ mice and comparable to Lyn^kd/kd^ mice (Fig. 6F). Paradoxically, however, despite similar IFN-I levels in Lyn^kd/kd^ and Lyn^−/−^ mice, spleen ISG expression was abrogated in aged Lyn^kd/kd^ mice compared to Lyn^−/−^ mice (Fig. 6F and fig. S15A).

These findings suggested that kinase-dead Lyn does not interfere with IFN-I production but may inhibit IFN-I responsiveness. To investigate further, the response of Lyn^−/−^ and Lyn^kd/kd^ cells to IFN-α was assessed. WT splenocytes showed robust phospho–signal transducer and activator of transcription 1 (STAT1) induction to IFN-α stimulation; however, induction was repressed in both Lyn^−/−^ and Lyn^kd/kd^ splenocytes, particularly in macrophages and neutrophils (Fig. 6G and fig. S15B). Whereas ISG induction to IFN-α occurred in all genotypes, ISG expression was suppressed in Lyn^kd/kd^ splenocytes and BMDMs compared to Lyn^−/−^ and WT cells (Fig. 6H and fig. S15, C and D). Collectively, these results suggest that Lyn regulates IFN production by promoting STAT1 phosphorylation via kinase-dependent mechanisms but inhibits signaling and downstream ISG transcription through kinase-independent processes.

### LynKD mice have attenuated peripheral T cell activation, correlating with unaltered thymocyte development

**Fig. 7.**
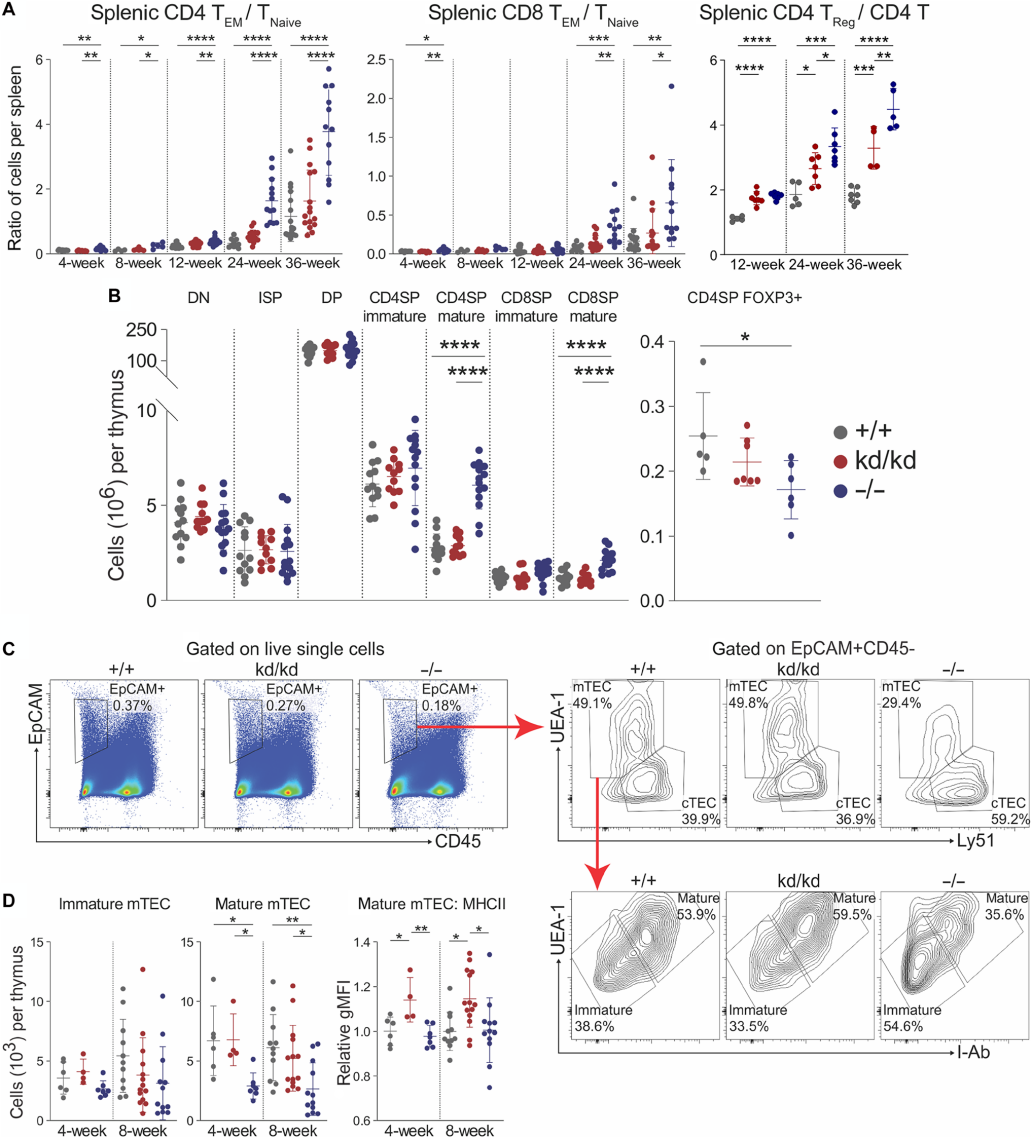
Lyn^kd/kd^ mice have attenuated peripheral T cell activation, correlating with normalized thymocyte development and mature mTEC numbers. (**A**) Left: Ratio of effector CD4 T cells to naïve CD4 T cells. Middle: Ratio of CD8 T effector cells to naïve CD8 T cells. Right: Ratio of CD4 T regulatory cells to total CD4 T cells, enumerated by flow cytometry of splenocytes, data compiled from 14 independent experiments (*n* = 4 to 21 Lyn^+/+^, 4 to 20 Lyn^kd/kd^, and 5 to 22 Lyn^−/−^ mice per time point). (**B**) Left: Thymocyte developmental populations enumerated by flow cytometry from 4-week-old mice. DN, double-negative; ISP, immature single-positive; DP, double-positive; SP, single-positive. Right: CD4SP FOXP3+ thymocytes from 8-week-old mice. Data compiled from three independent experiments (*n* = 5 to 12 Lyn^+/+^, 7 to 14 Lyn^kd/kd^, and 6 to 14 Lyn^−/−^ mice per time point). (**C**) Thymic epithelial cell flow cytometry gating strategy and (**D**) numbers of immature and mature mTECs and MHCII expression (relative gMFI) of mature mTECs from mice at the indicated ages, data compiled from three independent experiments (*n* = 6 to 11 Lyn^+/+^, 4 to 14 Lyn^kd/kd^, and 7 to 12 Lyn^−/−^ mice per time point). *P < 0.05, **P < 0.01, ***P < 0.001, and ****P < 0.0001 by one-way ANOVA with Holm-Šídák’s multiple comparisons test.

As with other spontaneous mouse models of lupus, autoimmune pathology in Lyn^−/−^ mice is T cell dependent, with T cells driving autoreactive B cell class switching to pathogenic isotypes (*24*). Given that mature conventional T cells do not express Lyn, T cell anomalies in Lyn^−/−^ mice are attributed to activation resulting from hyperactive cDCs and the inflammatory milieu (*3*). Consistent with previous reports (*8*, *30*), Lyn^−/−^ mice showed a skewing of CD4 and CD8 T cells toward effector memory (T_EM_) differentiation and away from naïve cells, tracking with disease progression (Fig. 7A). An increased proportion of CD4+ regulatory T cells (T_reg_ cells) in Lyn^−/−^ mice was also observed (Fig. 7A), which is thought to occur due to uncontrolled inflammation (*50*). In line with attenuated disease, Lyn^kd/kd^ mice showed a normal ratio of T_EM_ to T_Naive_ and a reduced frequency of CD4+ T_reg_ cells and CD4+CD69+ T cells compared to Lyn^−/−^ mice (Fig. 7A and fig. S16, A to C). Furthermore, although Lyn^−/−^ mice showed an expansion γδT17 cells with age, this was not observed in Lyn^kd/kd^ mice (fig. S16, A and C).

The finding of effector T cell differentiation as early as 4 weeks of age in Lyn^−/−^ mice was unexpected, being far earlier than previously reported and before an inflammatory milieu is established (*7*). Given that no differences were found between Lyn^kd/kd^ and Lyn^−/−^ cDCs in the periphery, which are the key population responsible for T cell priming, perturbations in T cell development were investigated as an alternative driver of early T cell activation in Lyn^−/−^ mice. Assessment of thymocytes at 4 weeks of age revealed that Lyn^−/−^ mice had a substantial expansion of mature CD4 single-positive (SP) and CD8SP thymocytes post–negative selection compared to both Lyn^kd/kd^ and WT mice (Fig. 7B and fig. S17A), suggestive of a deficit in the deletion of autoreactive T cells during development (*51*, *52*). The expanded CD4SP thymocyte population did not appear to differentiate into thymic T_reg_ cells in Lyn^−/−^ mice as CD4SP FOXP3+ thymocytes were slightly reduced compared to WT and Lyn^kd/kd^ mice (Fig. 7B and fig. S17A). A similar expansion in the proportion of mature CD4SP and CD8SP thymocytes was also observed in 8-week-old Lyn^−/−^ mice, although numbers were unchanged (fig. S17B). Lyn^−/−^ and Lyn^kd/kd^ thymus weights were also comparable to WT at both 4 and 8 weeks of age (fig. S17B), and no substantive differences were found at the double-negative (DN) or double-positive (DP) stages of development where the processes of beta selection and positive selection take place, respectively (*53*) (Fig. 7B and fig. S17C). The data therefore indicate that the SP negative selection checkpoint is the principal stage affected by Lyn deficiency.

Negative selection of autoreactive thymocytes is mediated through self-antigen presentation by mature medullary thymic epithelial cells (mature mTECs) and thymic antigen-presenting cells (APCs) including B cells, DCs, and macrophages (*54*). Thymic APCs in Lyn^−/−^ and Lyn^kd/kd^ mice were similar in number to controls except for a reduction in B cells and small increases in macrophages and cDC2s in Lyn^−/−^ mice, with APC MHCII expression also being comparable between Lyn^−/−^ and Lyn^kd/kd^ mice (fig. S18, A and B). Instead, Lyn^−/−^ mice were found to have a specific deficit in mature mTECs compared to WT and Lyn^kd/kd^ mice (Fig. 7, C and D). Immature mTEC numbers were unaffected, as were cortical thymic epithelial cell (cTEC) numbers (Fig. 7D and fig. S18C), indicating that Lyn deficiency specifically perturbs the maturation of mTECs. In contrast, Lyn^kd/kd^ mature mTECs were normal in number but exhibited elevated MHCII expression relative to Lyn^−/−^ and WT mature mTECs (Fig. 7D), suggesting enhanced antigen presentation and therefore implying enhanced negative selection in Lyn^kd/kd^ mice. These findings suggest Lyn-deficient mice may have a break in T cell central tolerance that likely contributes to peripheral T cell activation, implicating the kinase-independent functions of Lyn in mTEC maturation and maintenance of T cell central tolerance.

## DISCUSSION

This study aimed to delineate the kinase-dependent and kinase-independent functions of Lyn in SLE-like disease. Mice expressing kinase-dead Lyn were found to have mild and delayed autoinflammatory pathology compared to the more rapid and severe disease of Lyn-deficient mice, including limited IC-mediated glomerulonephritis, inflammatory cytokine induction, extramedullary hematopoiesis, and splenomegaly. This demonstrates that Lyn has critical inhibitory kinase-independent functions that complement Lyn’s kinase activity to maintain immune cell signaling balance and prevent SLE-like disease. Mechanistically, pathways that were rescued in Lyn^kd/kd^ mice, and thus are predominantly regulated by Lyn’s kinase-independent functions, included TLR pathway inhibition in B lymphocytes and monocytes/granulocytes, as well as the developmental program of MHCII^high^ mTECs. In contrast, the pathways that were perturbed in both Lyn^−/−^ and Lyn^kd/kd^ mice alike, and thus are predominantly regulated by the kinase activity of Lyn, included pDC inhibition of TLR-induced IFN-α production, cDC inhibition of LPS-induced IL-6 production, peripheral DC homeostasis, and inhibition of BCR cross-linking in B lymphocytes. In addition, Lyn was found to play a dual role in regulating the IFN-I signaling pathway by promoting STAT1 phosphorylation through its kinase activity while suppressing downstream ISG transcription through kinase-independent actions. Collectively, the results indicate that Lyn restrains SLE-like disease, in part, through its kinase-independent functions that include attenuating TLR pathway activation in B lymphocytes and myeloid cells, suppressing immune cell responsiveness to IFN-I, and maintaining normal thymocyte development.

Overall, minimal differences were identified in DCs between Lyn-deficient mice and Lyn^kd/kd^ mice, demonstrating that the kinase activity of Lyn is essential to DC homeostasis and inhibition. This probably reflects a loss of inhibitory ITIM-containing receptor phosphorylation as similar DC dysregulation is observed upon deficiencies of the Lyn targets PIR-B (*55*–*57*), FcγRIIb (*58*–*60*), and the putative Lyn target SiglecH in pDCs (*61*–*63*). Work in other lupus-prone mouse models has shown that hyperproduction of IL-6 and IFN-α by cDCs and pDCs, respectively, are critical in driving lupus-like disease (*64*–*66*). Therefore, it is likely that hyperactivity in the DC compartment contributes to disease in both the Lyn^kd/kd^ mice and Lyn^−/−^ mice.

In contrast to the negligible difference in DCs between Lyn^kd/kd^ and Lyn^−/−^ mice, Lyn^kd/kd^ B cells exhibited a markedly reduced propensity to produce cytokines to TLR agonism compared to Lyn^−/−^ B cells. B cell TLR inhibition is likely critical to the restraint of disease in Lyn^kd/kd^ mice, as in Lyn^−/−^ mice, MyD88 deficiency in either B cells or DCs is sufficient to attenuate disease although by different mechanisms; DC-MyD88 deficiency in Lyn^−/−^ mice abrogates inflammatory cytokine induction, whereas B cell–MyD88 deficiency attenuates autoreactive B cell class switching to reduce total IgG and anti-dsDNA IgG titers (*24*). Lyn^kd/kd^ mice showed features of disease attenuation consistent with B cell intrinsic TLR inhibition rather than DC inhibition as cytokine levels were not universally suppressed, with increases observed in *IL6*, *IFN*α, and *IFN*β. Instead, Lyn^kd/kd^ mice displayed attenuated IgG hyperglobulinemia, IgG ANA production, and ASC differentiation, echoing the findings of B cell MyD88-deficiency on the Lyn^−/−^ background (*24*). Other B cell defects of Lyn^−/−^ mice, such as B cell lymphopenia, BCR hyperactivity, and IgM hyperglobulinemia, were conserved in Lyn^kd/kd^ mice. This result echoes the findings from the B cell MyD88-deficient Lyn^−/−^ mouse model, where B cell defects persisted despite attenuated disease, including lymphopenia, plasmacytosis, IgM hyperglobulinemia, and elevated B cell costimulatory marker expression (*2*, *24*). The present study also uncouples these B cell compartment defects from the development of severe autoimmune disease, highlighting the principal importance of autoreactive B cell class switching to engender disease. Moreover, the findings extend previous works by delineating the kinase function of Lyn as inhibiting BCR signaling, whereas the kinase-independent function of Lyn inhibits the synergistic activation of the TLR pathway and putatively IRF5 in B cells, thereby suppressing autoreactive B cell class switching to maintain peripheral tolerance.

This study also identified a perturbation in T cell central tolerance processes in Lyn-deficient mice, an area that has not been previously investigated. Lyn^−/−^ mice were found to have an expansion of mature SP thymocytes and a coinciding deficit in mature MHCII^high^ mTECs. Mature mTECs induce the deletion of autoreactive SP thymocytes via the presentation of tissue-restricted antigens by MHC molecules, occurring through the promiscuous gene expression mediated by the autoimmune regulator (AIRE) (*54*). A deficit in negative selection caused by mTEC depletion or mTEC MHCII deficiency results in the expansion of mature SP thymocytes (*51*, *52*), suggesting a causal relationship between the loss of MHCII^high^ mTECs in Lyn^−/−^ mice and the expansion of SP mature thymocytes. Given that immature mTEC and cTEC numbers were unchanged in Lyn^−/−^ mice indicates that Lyn is specifically involved in the process of mature mTEC development and maintenance. This is supported by a previous study that found that the *Lyn* gene was differentially methylated in mature mTECs compared to immature mTECs (*67*). Although Lyn expression in epithelial cells is markedly lower than in immune cells, we and others have shown that Lyn plays a crucial role in epithelial cell signaling (*68*, *69*).

Another unexpected finding of this study was the role of kinase-dead Lyn in inhibiting ISG transcription in response to IFN-I. Investigating the IFN-I pathway revealed that both Lyn^−/−^ and Lyn^kd/kd^ macrophages and neutrophils had a lowered propensity for IFN-α–induced STAT1 phosphorylation, suggesting that Lyn kinase activity promotes STAT1 phosphorylation in this pathway. Although not examined here, this is likely initiated via phosphorylation of Janus kinase (JAK) as studies have reported that Lyn promotes JAK1 phosphorylation and ISG expression to dsDNA stimulation in BJAB cells (*70*), whereas in renal adenocarcinoma cells, Lyn drives JAK2 and STAT1 phosphorylation following IFN-γ–induced up-regulation of Lyn (*71*). Lyn kinase activity did not affect IFN-α–induced STAT1 phosphorylation in cDCs or pDCs, suggesting that this mechanism is cell type specific, which is consistent with a previous study showing that SFK inhibitors do not affect IFNβ-induced STAT1 phosphorylation in a human pDC cell line (*72*). Lyn^kd/kd^ BMDMs and splenocytes were found to have suppressed IFN-α–induced ISG transcription compared to both WT and Lyn^−/−^ cells. This is almost certainly due to a failure to activate the JAK-STAT pathway in response to IFN-α due to defective kinase activity while maintaining the capacity to inhibit ISG transcription through kinase-independent mechanisms. Such governance is lost in Lyn-deficient cells, and together with the cell type–specific nature of this regulation, this likely explains why ISG expression was still observed in Lyn^−/−^ samples. Thus, our data implicate Lyn as regulating IFN-I signaling by promoting STAT1 phosphorylation through Lyn’s kinase activity while simultaneously inhibiting downstream ISG transcription through kinase-independent protein-protein interactions. This is likely by binding IRF9 in this pathway to prevent the formation of the heterotrimeric ISGF3 complex of STAT1-STAT2-IRF9 and its nuclear localization to induce ISG transcription (*73*). Collectively, although Lyn^kd/kd^ mice do not have restrained IFN-α hyperproduction, they demonstrate inhibited IFN-I responsiveness that likely contributes to disease attenuation.

**Fig. 8.**
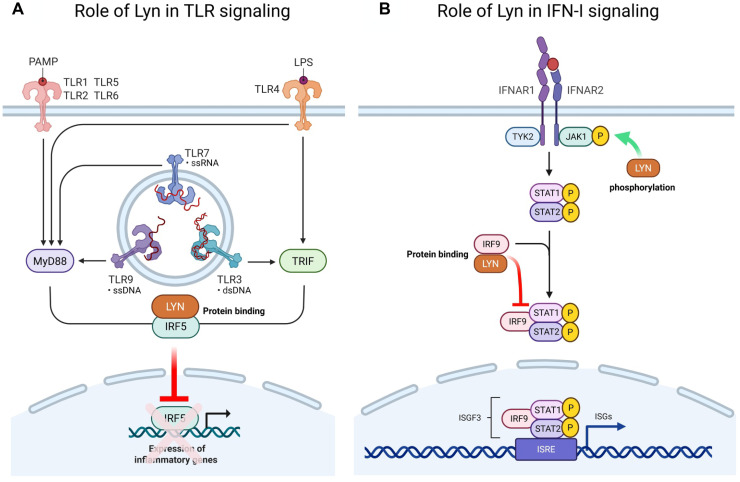
Proposed kinase-dependent and kinase-independent functions of Lyn in regulating the TLR and IFN signaling pathways. On the basis of our data and that of others (*22*, *25*), we propose that (**A**) Lyn inhibits cytokine production in the TLR pathway independent of kinase activity in B lymphocytes, monocytes, and granulocytes, consistent with Lyn binding to IRF5 and preventing its nuclear localization. (**B**) In the IFN-I signaling pathway, Lyn promotes JAK-STAT1 phosphorylation through its kinase activity while inhibiting the transcription of ISGs through its kinase-independent activity, which is consistent with an inhibitory binding interaction with IRF9 to prevent the formation of the ISG3 complex. Created in BioRender. Hibbs, M. (2025) https://BioRender.com/2mpgbgr.

A major strength of our study is the generation of mice harboring a bona fide kinase-dead mutation of Lyn. This has allowed us to begin to define the kinase-independent functions of Lyn in vivo, whereas previous research has only made use of transfected or mutated cell lines (*20*–*22*). We have shown that pathways regulated by kinase-dead Lyn include the TLR pathway in B cells and myeloid cells, the IFN-I pathway in immune cells, and the developmental program of mTECs. However, a limitation of our study is that we have not yet shown biochemically how kinase-dead Lyn mediates its actions. Previous reports in the literature demonstrate interactions between Lyn and the IRF family of transcription factors (*22*, *25*, *74*). Our data favor a view that Lyn suppresses the TLR pathway by inhibiting IRF5 via protein-protein interactions (Fig. 8A), consistent with the previously described mechanism (*22*), particularly in B lymphocytes and myeloid cells. Similarly, we propose that Lyn may also inhibit IRF9 through protein-protein interactions to suppress immune cell ISG transcription in response to IFN-I stimulation (Fig. 8B). However, the inhibition of these pathways may occur in unison with Lyn phosphorylating the IRFs (*22*, *25*), with the dominance of each mechanism being cell type and context specific, highlighting the cell type–specific nature of Lyn’s functions and mechanisms of action (*38*). Supporting a role for IRF interactions in the kinase-independent functions of Lyn, we note that the features of the Lyn^kd/kd^ B cell compartment mirror the findings of B cell IRF5 deficiency in other lupus-prone mouse models. B cell monoallelic IRF5 deficiency in the FcγRIIB^−/−^Yaa lupus model also restrains B cell IL-6 and TNFα production to R848 and CpG-B stimulation ex vivo and lowers IgG isotype titers in vivo, as well as IgG ANAs and ASC numbers, leading to attenuated disease (*75*). Similar findings were reported in the pristane-induced lupus model, where autoreactive B cell class switching to pathogenic IgG was dependent on IRF5 (*76*, *77*). Lyn^kd/kd^ Fo B cells also demonstrated a pronounced up-regulation of surface IgD, another feature of IRF5 knockdown in B cells (*78*). Lyn’s protein-protein interactions in the IRF pathway may also be implicated in mTEC development where the frequencies of mTECs and thymocytes are altered in Lyn^−/−^ mice but normal in Lyn^kd/kd^ mice. mTEC development depends on tonic IRF (*79*) and TRAF6 signaling (*80*), and a kinase-dead Lyn variant has been demonstrated to bind IRF5 and block TRAF6 in vitro (*22*).

Overall, the work presented here demonstrates critical kinase-independent functions of Lyn in vivo that are both wide-ranging and cell type specific. Collectively, this work extends our knowledge of the pathways regulated by Lyn and the nature of this regulation, with further research having the potential to uncover therapeutic targets for the treatment of lupus. Furthermore, additional work is required to investigate the signaling pathways perturbed in Lyn-deficient mTECs and to define the protein-protein interaction used by Lyn. Although IRFs are postulated to be inhibitory targets of kinase-dead Lyn, using immune cell samples from Lyn^kd/kd^ mice, we can now use unbiased approaches such as coimmunoprecipitation-coupled mass spectroscopy to find alternative substrates that occur in vivo, which would enhance the findings from this study.

## MATERIALS AND METHODS

### Experimental design

The focus of this study was to investigate the kinase-independent functions of Lyn in autoimmune disease development, and thus, studies primarily used mice that had been engineered to carry a mutation in the ATP-binding site of Lyn (Lyn^Kd/Kd^). Autoimmune disease progression in Lyn^kd/kd^ mice was compared to that of Lyn-deficient mice, with C57BL/6 mice used as controls. Tissue and immune cell samples from mice underwent various in vivo, ex vivo, and in vitro experiments, using biological and technical replicates. Mice were age and sex matched, samples were collected across multiple experiments and study endpoints, and data were pooled. Details on the statistical tests used and group sizes used are presented in the figure legends.

### Mice

Lyn kinase-dead mutant mice on the C57BL/6 background (Lyn^kd/kd^) were generated by targeted homologous recombination using the services of Ozgene Pty Ltd. (Bentley, WA, Australia). The targeting construct contained a point mutation in exon 9 of the active *Lyn* gene, altering the critical ATP-binding residue lysine to a methionine (p.K275M), as well as a floxed PGKNeo transcription cassette to aid in selection of homologous recombinants (fig. S1A). C57BL/6 embryonic stem cells were electroporated and Southern blotting was performed to screen for homologous recombination events. Inbred C57BL/6 background mice were generated from germline transmitting chimeras, and the floxed selection cassette was excised using EIIa-Cre deleter mice (*81*) (JAX stock no. 003724). The absence of Lyn kinase activity was confirmed in an in vitro kinase assay (Fig. 1A).

C57BL/6 background Lyn^−/−^ mice have been described previously (7). C57BL/6 control mice (Lyn^+/+^; WT) were purchased from Monash Animal Services (Clayton, Australia). Mice were housed in specific pathogen–free conditions at the Monash Animal Research Platform (Monash University, Clayton, Australia) and Monash Intensive Care Unit (Alfred Research Alliance, Melbourne, Australia) in Optimice cages and were age and sex matched for experiments. To assess the impact of environmental inflammatory challenge on phenotypes, a cohort of mice was also aged in a low-barrier facility in open-top cages (LAF-13F, Monash University, Clayton, Australia) (fig. S2). All experiments were performed in accordance with the ARRIVE guidelines and the National Health and Medical Research Council guidelines for animal experimentation. Experiments were approved by the Animal Ethics Committee of the Alfred Research Alliance (project nos. E1688-2016M and E8228-2021M).

### Flow cytometry

Single-cell suspensions were prepared from spleens by extrusion, BM by flushing femurs, and lymph nodes by enzymatic digestion for 30 min at room temperature [Liberase TL (0.1 mg/ml; Roche Diagnostics, North Ryde, NSW, Australia), DNAse I (0.13 mg/ml; Roche), 10% (v/v) fetal bovine serum (FBS) (Bovogen Biologicals, East Keilor, VIC, Australia), penicillin-streptomycin (100 IU/ml; Gibco│Thermo Fisher Scientific, Scoresby, VIC, Australia), and RPMI 1640 growth medium (Gibco)]. Thymi were mechanically dissociated by passing through a 70-μm sieve to obtain a thymocyte fraction, with the unextruded thymic fragments collected and digested for 30 min at 37°C to obtain an epithelial cell–enriched fraction [Liberase (0.05 mg/ml), DNAse I (0.13 mg/ml), 10% FBS, penicillin-streptomycin (100 IU/ml), and RPMI]. Blood was harvested by cardiac puncture into EDTA-containing tubes, and volume was recorded before centrifuging to remove the plasma. For kidneys, mice were transcardially perfused with saline and kidneys were collected and digested at 37°C for 50 min [Liberase (0.1 mg/ml), DNAse I (0.1 mg/ml), 10% FBS, penicillin-streptomycin (100 IU/ml), 50 μM 2-mercaptoethanol, and 2 mM GlutaMAX (Gibco)]. Red blood cells were removed using ammonium-chloride-potassium lysis buffer, and single-cell suspensions were filtered and counted using the Z2 Coulter Counter (Beckman Coulter, Mount Waverley, VIC, Australia) except for kidney cells, which were enumerated using flow cytometry cell counting beads (BD CaliBRITE, BD Biosciences, Macquarie Park, NSW, Australia). Cells were incubated for 5 to 10 min with anti-CD16/CD32 to block Fc receptors and surface stained for 30 min on ice with biotinylated or fluorophore-conjugated antibodies (file S1). Intracellular FoxP3 staining was performed using the Foxp3/Transcription Factor Staining Buffer Set (eBioscience, Waltham, MA, USA). For intracellular cytokine staining, cells were fixed with paraformaldehyde, permeabilized with BD Perm/Wash (BD Biosciences), and stained with monoclonal antibodies to IL-12p40, IL-6, and TNFα or polyclonal rabbit anti-mouse IFN- and goat anti-rabbit IgG-AF488 secondary. Staining was also performed with IgG1κ isotype controls (MOPC-21), and anti-cytokine staining of unstimulated samples was used to set the gate for cytokine-positive cells. For phospho-STAT1, cells were fixed for 10 min at 37°C with BD Cytofix Fixation Buffer (BD Biosciences), surface stained with antibodies to B220, CD11b, CD11c, F4/80, I-A/I-E, Ly-6G, and PDCA1 (file S1), permeabilized with BD Phosflow Perm Buffer III (BD Biosciences), and stained with anti–phospho-STAT1-Y701. Anti–phospho-STAT1 staining of unstimulated samples was used as the negative control. LIVE/DEAD Fixable Aqua Dead Cell Stain (Invitrogen) was used to exclude dead cells in fixed preparations and FluoroGold in nonfixed preparations. Flow cytometry data were acquired on the BD LSRFortessa X-20 and analyzed using FlowJo software (BD Biosciences). Expression of cell surface markers was determined by geometric mean fluorescence intensity (gMFI) and normalized to C57BL/6 mice for each experiment (relative gMFI) to allow data to be pooled.

### BMDM cell culture and ex vivo splenocyte stimulation

For intracellular cytokine staining, splenocytes from young (4- to 6-week-old) mice were aseptically seeded in tissue culture–treated culture plates with media [2% (w/v) bovine serum albumin (BSA), penicillin-streptomycin (100 IU/ml), BD GolgiPlug (BD Biosciences), and RPMI] and stimulated for 4 hours at 37°C with R848 (resiquimod; 5 μg/ml), Poly(I:C) (5 μg/ml), 1 μM CpG-C ODN 2395 (Miltenyi Biotec, Macquarie Park, NSW, Australia), or LPS (5 μg/ml; Sigma-Aldrich│Merck, Bayswater, VIC, Australia). Cells were harvested using cold Dulbecco’s phosphate-buffered saline (PBS) without calcium or magnesium (Gibco) and 5 mM EDTA. For phospho-STAT1 flow cytometry and ISG real-time polymerase chain reaction (RT-PCR), splenocytes from young mice were stimulated with mouse IFN-α (1000 IU/ml; Miltenyi Biotec) in RPMI for 30 min or 8 hours, respectively. For BMDMs, femurs and tibias were aseptically flushed, and cells were seeded in nontissue culture–treated Petri dishes with media [15% FBS, 20% conditioned media from mouse L cells (American Type Culture Collection, catalog no. CRL-2648), 2 mM GlutaMAX, penicillin-streptomycin (100 IU/ml), and high-glucose Dulbecco’s modified Eagle’s medium (Gibco)]. Cells were supplemented with L cell conditioned media on day 3 and day 5 and harvested on day 6 using TrypLE (Gibco), hematocytometer counted, and replated in Dulbecco’s modified Eagle’s medium overnight before stimulation.

### B cell purification and proliferation assay

Splenic B cells were purified from young mice (>95% purity) by negative selection on MACS columns (Miltenyi Biotec) and stimulated with 10 μg/ml F(ab′)_2_ goat anti-mouse IgM, intact goat anti-mouse IgM (10 μg/ml; Jackson ImmunoResearch Laboratories, West Grover, PA, USA), or medium for 72 hours. [^3^H]Thymidine was added for the final 6 hours, and cells were harvested using an automated cell harvester (TomTec, Unterschleissheim, Germany) and analyzed with a scintillation counter (Packard Instruments) to assess proliferation as previously described (*18*).

### Lyn kinase activity assay

BMDMs were lysed with 100 mM NaCl, 10 mM tris-HCl (pH 7.5), 1% NP-40, 2 mM EDTA, 0.1 mM pervanadate, 10 mM MgCl_2_, and cOmplete Protease Inhibitor Cocktail (Roche) (*82*). A 100-μg protein lysate was precleared with protein A–Sepharose slurry, and Lyn was then immunoprecipitated using anti-Lyn antibody (Santa Cruz Biotechnology, Dallas, TX, USA) and protein A–Sepharose as previously described (*82*). The amount of Lyn present in each sample was determined by eluting immunoprecipitated Lyn from beads followed by electrophoresis and immunoblotting with anti-Lyn (Santa Cruz Biotechnology) to equalized Lyn levels in WT and Lyn^kd/kd^ samples on the basis of densitometry. After washing beads, immunoprecipitated Lyn was incubated for 15 min at 30°C with 25 mM Tris (pH 7.5), 0.5 mM dithiothreitol, 0.1 mM pervanadate, 50 μM cold ATP, 10 mM MgCl_2_, 0.25 ml of [γ-^32^P]ATP (specific activity, 2800 cpm/pmol), and 5 μM Src-optimal peptide (SOP; AEEEIYGEFEAKKKK) (*82*). The reaction was terminated by centrifugation followed by spotting of the supernatant onto Whatman P81 chromatography paper (Whatman, Clifton, NJ). Unincorporated [γ-^32^P]ATP was eliminated by a 10-min wash and two 5-min washes in 0.4% orthophosphoric acid followed by a 5-min wash in 100% ethanol. The phosphorylated SOP bound to the paper was immersed in beta counter scintillation fluid and counted with a beta counter (Packard Instruments) (*82*).

### Spleen progenitor assay

Spleen hematopoietic progenitors in aged (26- to 30-week-old) mice were quantitated using semisolid 0.3% agar cultures containing 100,000 splenocytes. Colony formation was stimulated by macrophage colony-stimulating factor (M-CSF) (10 ng/ml), IL-3 (10 ng/ml), or granulocyte-macrophage colony-stimulating factor (GM-CSF) (10 ng/ml) (PeproTech│Thermo Fisher Scientific). Colonies (>50 cells) were counted after 7 days as previously described (*10*).

### Immunoblotting

B cells and BMDMs were lysed in 1% Triton X-100, 0.1% SDS, 20 mM tris-HCl (pH 7.5), 150 mM NaCl, 2 mM EDTA, cOmplete Protease Inhibitor Cocktail (Roche), 1 mM pervanadate, and 50 mM NaF. Clarified lysates were subjected to electrophoresis and immunoblotting analysis using antibodies specific to Lyn (R355, rabbit polyclonal anti-Lyn made in-house), phospho-AKT (S473), AKT, phospho-ERK1/2 (T202/Y204), or ERK1/2 (Cell Signaling Technology, Danvers, MA, USA). Following incubation with fluorescent-conjugated secondary antibodies (LI-COR Biosciences, Mulgrave, VIC, Australia), membranes were scanned using the Odyssey infrared imaging system (LI-COR Biosciences) and converted to grayscale using Odyssey Application software version 2.0, with fluorescence signal quantified using ImageJ (Fiji).

### Real-time polymerase chain reaction

RNA was extracted from cultured cells or frozen tissue using TRIzol and reverse transcribed with the FIREScript RT cDNA Synthesis KIT (Solis BioDyne, Tartu, Estonia), with RT-PCR performed on the QuantStudio 6 Flex Real-Time PCR System using PowerUp SYBR Green Master Mix (Applied Biosystems│Thermo Fisher Scientific) and in-house validated probes (file S2). Samples were run in triplicate, and relative expression was determined using the ∆∆CT method, normalized to *18S* for RNA isolated from splenocytes and spleen tissue and *Gapdh* for BMDMs and kidney tissue. Splenocyte and BMDM ISG expression was calculated by averaging the expression of six to seven ISGs.

### Kidney histopathology

Saline-perfused kidneys were fixed in 10% neutral-buffered formalin for 24 hours, processed, and paraffin embedded. Sections (3 μm) were stained with periodic acid–Schiff (PAS), scanned using Aperio Scanscope AT Turbo, and analyzed with Aperio ImageScope software (Leica Biosystems, Nussloch, Germany). Outer cortical glomeruli (minimum of 50 per mouse) were analyzed by tracing the parietal layer of the Bowman’s capsule to determine glomerular cross-sectional area, and glomerular morphology was scored according to previously described criteria (*30*): 0, normal glomerular cellularity and morphology; 1, mild cellular expansion and/or early-stage disrupted glomerular morphology; 2, moderate cellular expansion with distinct disruption to glomerular morphology that includes lobularity and/or Bowman’s space enlargement; 3, advanced cellular expansion/consolidation with severe lobularity or fragmentation, segmental necrosis (patches of anuclear pink staining), and/or periglomerular cellular expansion; and 4, end-stage glomerular destruction, progressive or complete loss of cellularity, and/or distinct glomerular morphology (fig. S1D). The mean glomerular disease morphology score and cross-sectional area were taken for each mouse. All measurements were performed blinded.

### Immunofluorescence staining

Saline-perfused kidneys were fixed for 4 hours at 4°C in periodate-lysine-paraformaldehyde [0.22% (w/v) sodium periodate, 1.37% (w/v) l-lysine, 2% (w/v) paraformaldehyde, and PBS] and infused with 20% (w/v) sucrose solution before being snap frozen in Optimal Cutting Temperature compound (Sakura Finetek, Tokyo, Japan) with liquid nitrogen. Cryostat sections (7 μm) were cut onto Superfrost Plus slides, rehydrated in PBS, blocked with 4% (w/v) BSA, and stained with goat anti-mouse IgG-AF488 (InvivoGen, San Diego, USA) or goat anti-mouse complement C3-FITC (MP Biomedicals, Irvine, CA, USA) for 60 min at room temperature and then washed in PBS, mounted in antifade, and imaged using the Eclipse TE2000-U Inverted Fluorescence Microscope and DS-Ri2 camera (Nikon Australia, Rhodes, NSW, Australia). Fluorescence intensity was quantified using ImageJ (Fiji) by tracing outer cortical glomeruli (minimum of 35 glomeruli per mouse) using the polygon tool and measuring the mean gray value. All measurements were performed blinded.

### Enzyme-linked immunosorbent assay

Plasma anti-dsDNA IgG titers were determined by incubating plasma with calf thymus DNA-coated plates (Sigma-Aldrich│Merck) and using horseradish peroxidase (HRP)–conjugated goat anti-mouse IgG detection antibody (SouthernBiotech, Birmingham, AL, USA) as previously described (*30*). Relative titers were calculated by plotting the optical density against a standard curve of a high anti-dsDNA IgG reference sample (in-house), with titers normalized to C57BL/6 mice within each aging cohort to allow data from different experiments to be collated. BAFF levels in serum were measured using the Mouse BAFF/BLyS/TNFSF13B Quantikine enzyme-linked immunosorbent assay (ELISA) kit (R&D Systems, Minneapolis, MN, USA). Plasma Ig titers were determined using polyvalent goat anti-mouse Ig(H+L) capture antibody and HRP–conjugated anti-IgM, anti-IgG1, anti-IgG2b, anti-IgG2c, or anti-IgG3 detecting antibodies, with purified Ig used for the standard curve (SouthernBiotech).

### Statistical analysis

Statistical significance was assessed by one-way analysis of variance (ANOVA) with Holm-Šídák’s multiple comparisons test using GraphPad Prism software version 9.2 (GraphPad Software, Boston, MA, USA). Data with *P* values < 0.05 were considered statistically significant. Significance is indicated by the following: *P < 0.05, **P < 0.01, ***P < 0.001, and ****P < 0.0001. Bars on graphs represent means ± SD unless otherwise stated.

## Supplementary Materials

### The PDF file includes:

Figs. S1 to S18 Table S1 Legends for files S1 and S2 References
