# Supplementary material for: Lyn restrains lupus via kinase-independent mechanisms that limit Toll-like receptor activation and type I interferon responsiveness
Source: Sci Adv. 2025 Oct 17;11(42):eadz1726. doi: 10.1126/sciadv.adz1726 (PMC12533645; doi:10.1126/sciadv.adz1726)
Supplement: Supplementary file 1 — Figs. S1 to S18 Table S1 Legends for files S1 and S2 References [file sciadv.adz1726_sm.pdf]

Science Advances
AAAS

Supplementary Materials for

Lyn restrains lupus via kinase-independent mechanisms that limit Toll-like receptor activation and type I interferon responsiveness

Elan L'Estrange-Stranieri *et al.*

Corresponding author: Margaret L. Hibbs, margaret.hibbs@monash.edu

Sci. Adv. **11**, eadz1726 (2025)
DOI: 10.1126/sciadv.adz1726

The PDF file includes:

Figs. S1 to S18
Table S1
Legends for files S1 and S2
References

Other Supplementary Material for this manuscript includes the following:

Files S1 and S2

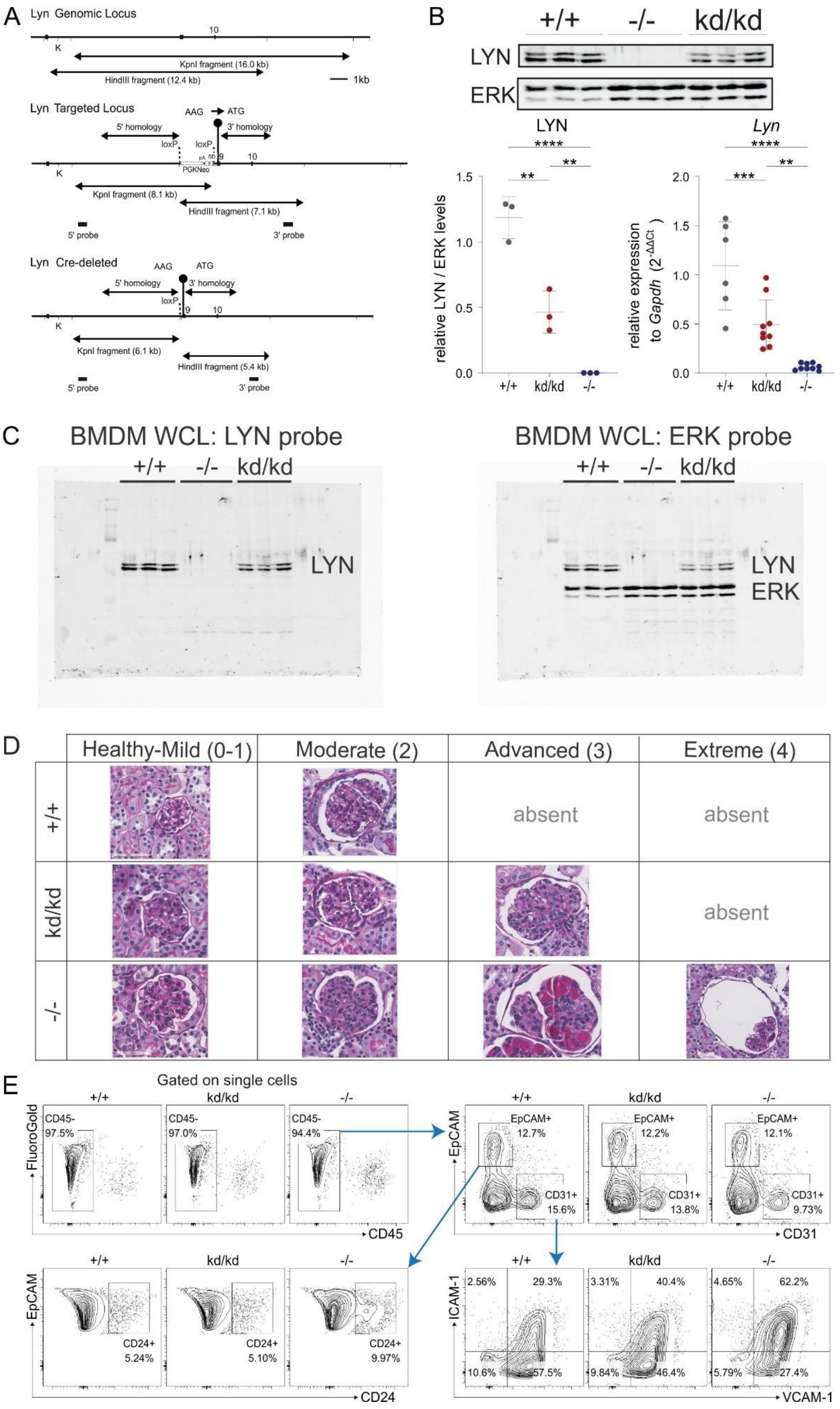
A
Lyn Genomic Locus
KpnI fragment (16.0 kb)
HindIII fragment (12.4 kb)
1kb
Lyn Targeted Locus
AAG → ATG
5' homology
3' homology
loxP
PGKNeo
KpnI fragment (8.1 kb)
HindIII fragment (7.1 kb)
5' probe
3' probe
Lyn Cre-deleted
KpnI fragment (6.1 kb)
HindIII fragment (5.4 kb)
B
+/+
-/-
kd/kd
LYN
ERK
relative LYN / ERK levels
relative expression to Gapdh (2^-ΔΔCt)
C
BMDM WCL: LYN probe
BMDM WCL: ERK probe
D
Healthy-Mild (0-1)
Moderate (2)
Advanced (3)
Extreme (4)
absent
E
Gated on single cells
FluoroGold
CD45
CD45- 97.5%
CD45- 97.0%
CD45- 94.4%
EpCAM
CD31
EpCAM+ 12.7%
EpCAM+ 12.2%
EpCAM+ 12.1%
CD31+ 15.6%
CD31+ 13.8%
CD31+ 9.73%
CD24
CD24+ 5.24%
CD24+ 5.10%
CD24+ 9.97%
ICAM-1
VCAM-1
2.56%
29.3%
10.6%
57.5%
3.31%
40.4%
9.84%
46.4%
4.65%
62.2%
5.79%
27.4%

Fig. S1. Generation of $Lyn^{kd/kd}$ mice and quantification of kidney histopathology. (**A**) Lyn genomic locus highlighting the location of exons 8-10. Middle: targeted Lyn locus depicting AAG (Lysine; K) to ATG (Methionine; M) point mutation in exon 9, location of LoxP sites flanking PGKNeo transcription cassette, 5' and 3' homology arms, relevant restriction fragments and screening probes. Bottom: Lyn locus following Cre-mediated excision of selection cassette. H = HindIII, K = KpnI. (**B**) Immunoblot of anti-LYN and anti-ERK (loading control) from BMDMs (n = 3 mice per genotype) and densitometry (bottom left), data representative of two independent experiments. Bottom right: Lyn levels determined by RT-PCR of spleen tissue from 36-week-old (n = 6 $Lyn^{+/+}$, 9 $Lyn^{kd/kd}$, 9 $Lyn^{-/-}$) mice. Horizontal lines on graphs indicate mean ± standard deviation. **P < 0.01, ***P < 0.001, ****P < 0.0001 by one-way ANOVA with Holm-Šídák's multiple comparisons test. (**C**) Uncropped Western blots of data in (B). LHS blot: LYN probe of whole cell lysate (WCL) prepared from BMDMs; RHS blot: ERK probe of unstripped blot, post-LYN probe. (**D**) Kidney glomerular disease morphology scoring system; examples of each grade shown from mice. (**E**) Flow cytometry gating strategy and representative plots of kidney endothelial and epithelial cell populations from 24-week-old mice.

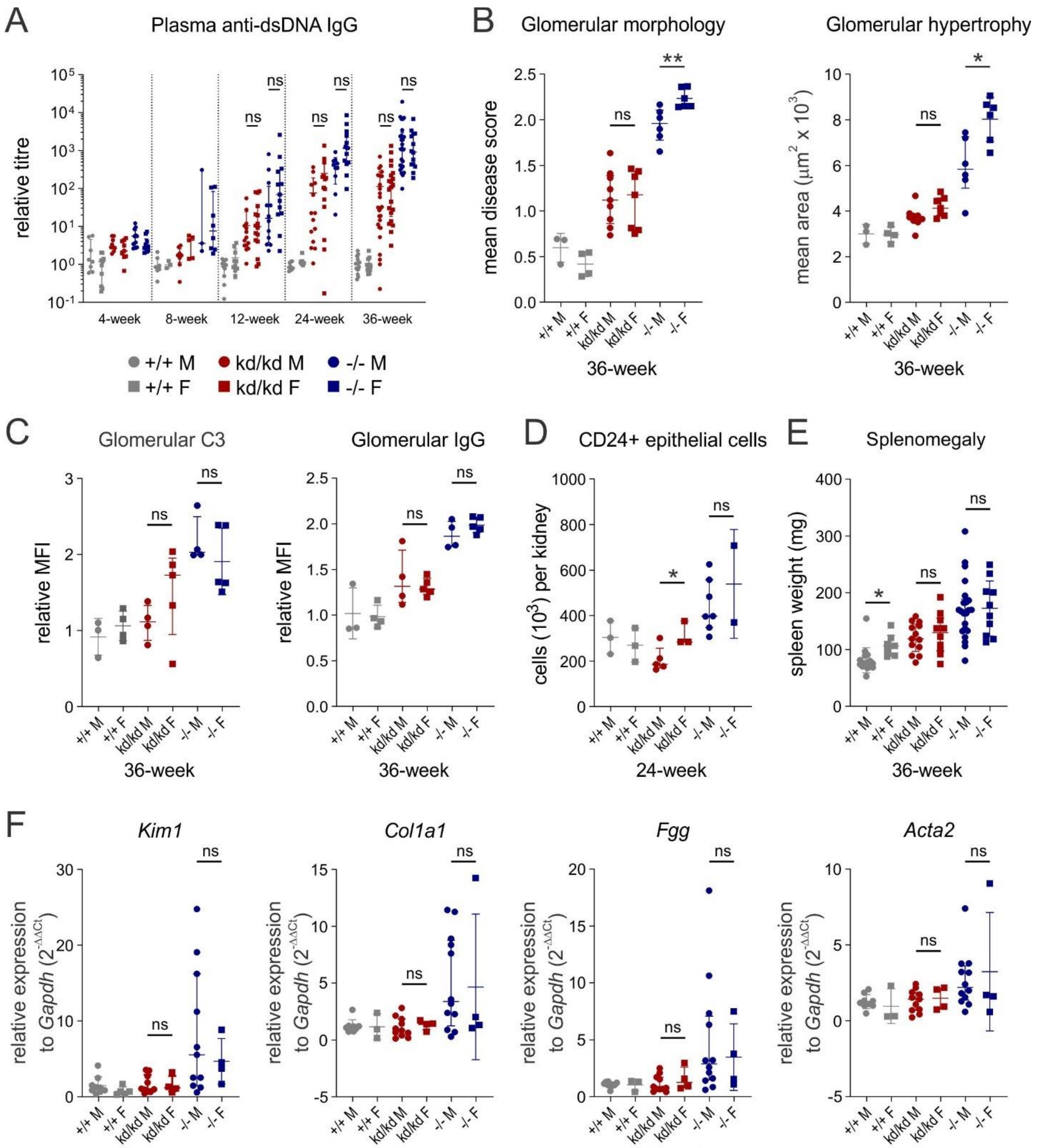

Fig. S2. Lyn$^{kd/kd}$ mice show no sex bias in disease parameters. Autoimmune disease parameters previously presented (**Fig. 1, B-E**) including (**A**) plasma anti-dsDNA IgG titers; (**B**) glomerular pathology; (**C**) glomerular C3 and IgG mean fluorescence intensity; (**D**) CD24+ epithelial cells in kidney; (**E**) spleen weight; and, (**F**) profibrotic gene expression in kidney were replotted to display the sex of the mice (round symbols = male, square symbols = female). Horizontal lines on graphs indicate mean ± standard deviation (B-F) or SEM (A). ns = not significant, **P* < 0.05, ***P* < 0.01, by unpaired t-test, comparing males versus females of each genotype.

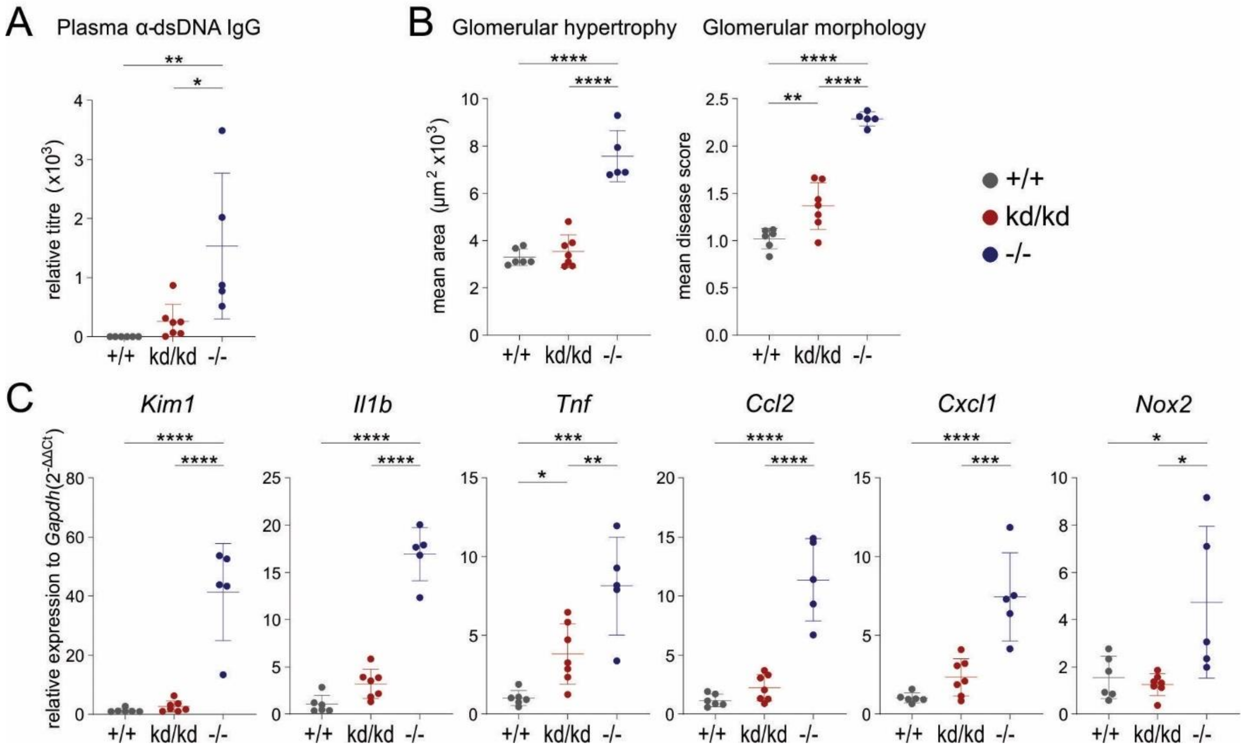

Fig. S3. Lyn$^{kd/kd}$ mice aged in a low barrier facility show restrained disease compared to Lyn-deficient mice. (**A**) Plasma anti-dsDNA IgG titre determined by ELISA. (**B**) Glomerular cross-sectional area and glomerular morphology disease scoring of PAS-stained kidney sections. (**C**) Gene expression in kidney tissue determined by RT-PCR. Mice were assessed at 30 weeks of age (n = 6 Lyn$^{+/+}$, 7 Lyn$^{kd/kd}$, 5 Lyn$^{-/-}$). Horizontal bars indicate mean ± SD. **P* < 0.05, ***P* < 0.01, ****P* < 0.001, *****P* < 0.0001 by one-way ANOVA with Holm-Šídák's multiple comparisons test.

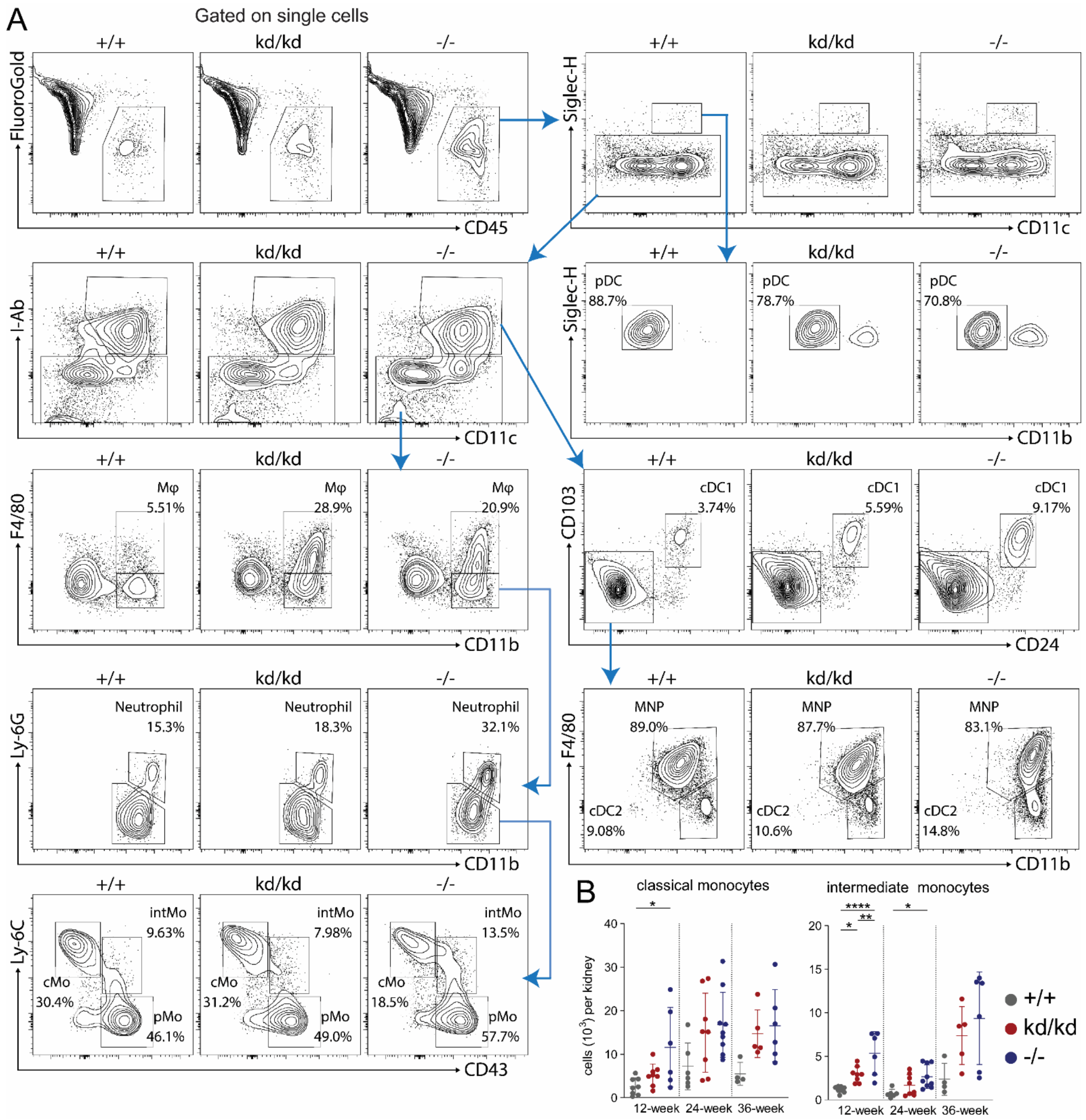

Fig. S4. Kidney myeloid cell flow cytometry gating strategy. (**A**) Representative plots from 36-week-old mice. Gating strategy adapted from Brähler et al (*36*). (**B**) Renal classical and intermediate monocytes enumerated by flow cytometry (n = 4 to 8 $Lyn^{+/+}$, 5 to 8 $Lyn^{kd/kd}$, 6 to 9 $Lyn^{-/-}$ mice) at the indicated ages, data compiled from five independent experiments. Horizontal bars indicate mean ± SD. **P* < 0.05, ***P* < 0.01, *****P* < 0.0001 by one-way ANOVA with Holm-Šídák's multiple comparisons test.

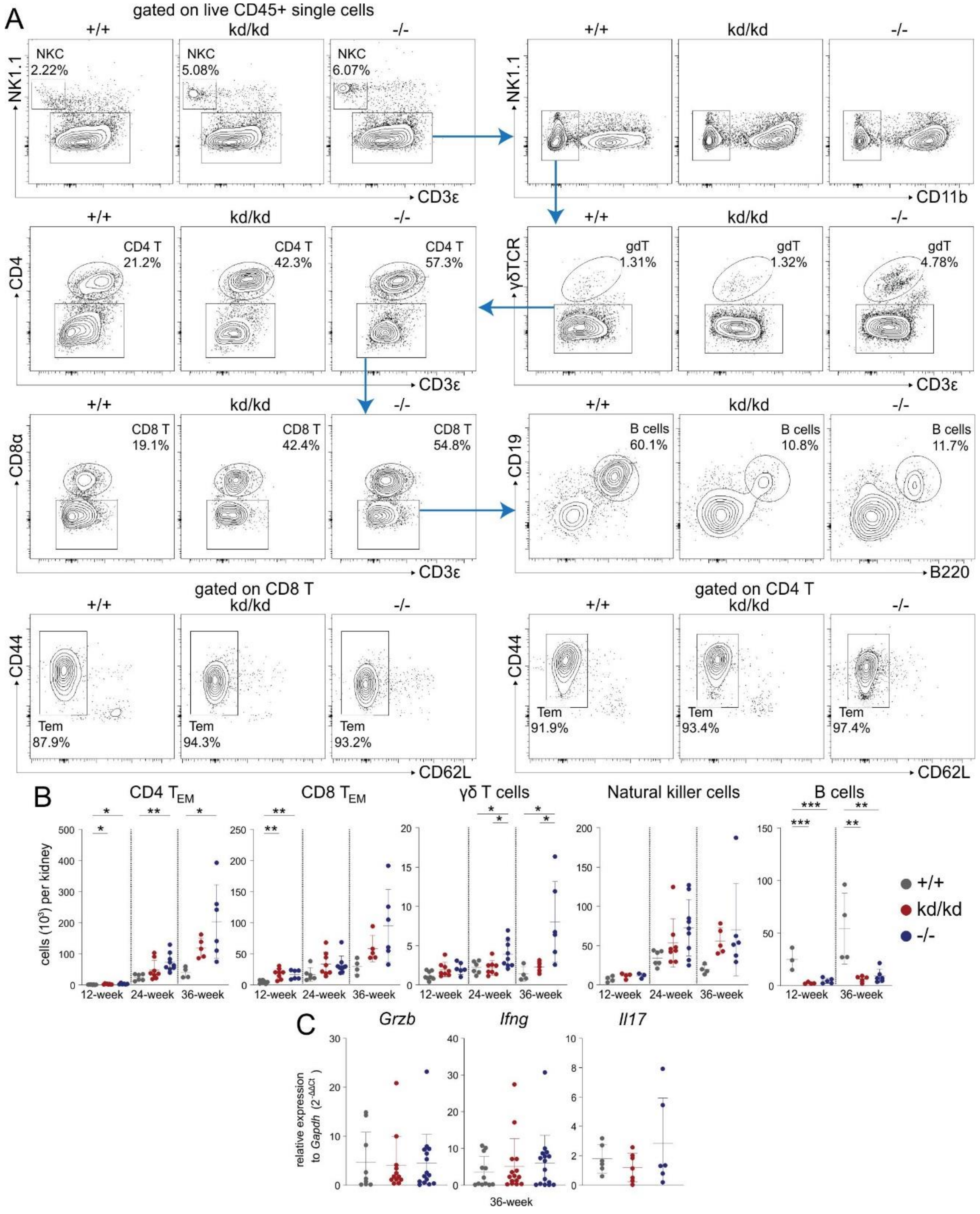

Fig. S5. Kidney lymphocyte flow cytometry gating strategy. (**A**) Representative plots from 36-week-old mice. Natural killer cells, NKC; γδ T cells, gdT; Effector memory, T_{EM}. (**B**) Renal lymphocytes enumerated by flow cytometry (n = 3 to 8 Lyn$^{+/+}$, 4 to 8 Lyn$^{kd/kd}$, 5 to 9 Lyn$^{-/-}$) mice at the indicated ages. (**C**) Expression of effector T cell genes determined by RT-PCR of kidney tissue from 36-week-old (n = 6 to 12 Lyn$^{+/+}$, 7 to 15 Lyn$^{kd/kd}$, 6 to 16 Lyn$^{-/-}$) mice. Horizontal bars indicate mean ± SD. *$P < 0.05$, **$P < 0.01$, ***$P < 0.001$ by one-way ANOVA with Holm-Šídák's multiple comparisons test.

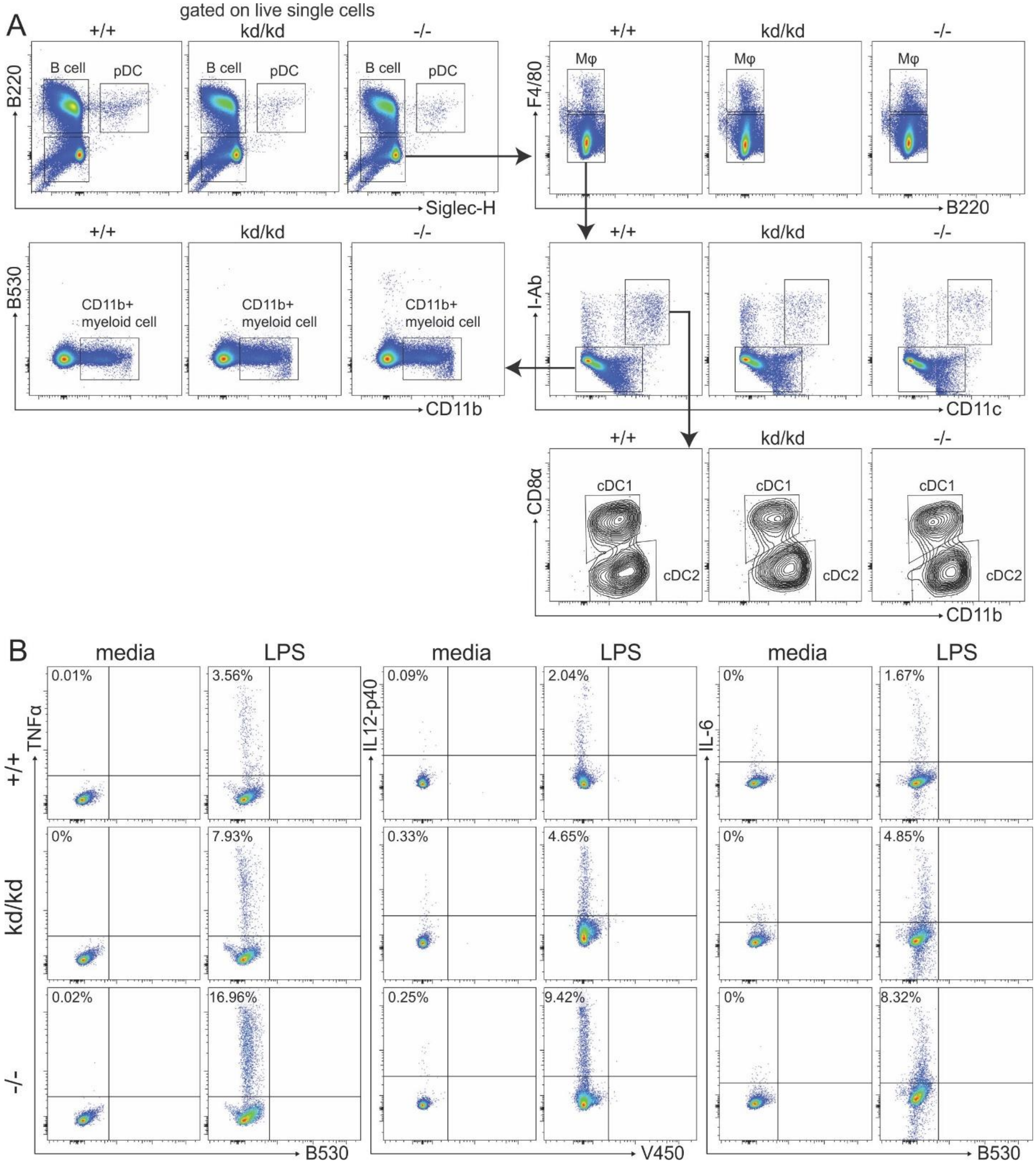

Fig. S6. Splenocyte intracellular cytokine flow cytometry. (**A**) Gating strategy used to define splenocyte subsets in 4-6-week-old mice. CD11b+ splenocytes (predominantly monocytes and granulocytes) were unable to be further subsetted as the M-CSFR was found to be downregulated following *ex vivo* culture at 37°C, consistent with previous reports (*83*). (**B**) Representative plots of CD11b+ splenocytes showing intracellular cytokine staining on the vertical axes and empty channels (B530 or V450) on the horizontal axes, from samples cultured with media or LPS in the presence of BD GolgiPlug for 4 hours. Staining controls were stimulated samples stained with isotype control antibodies, and anti-cytokine staining of unstimulated samples.

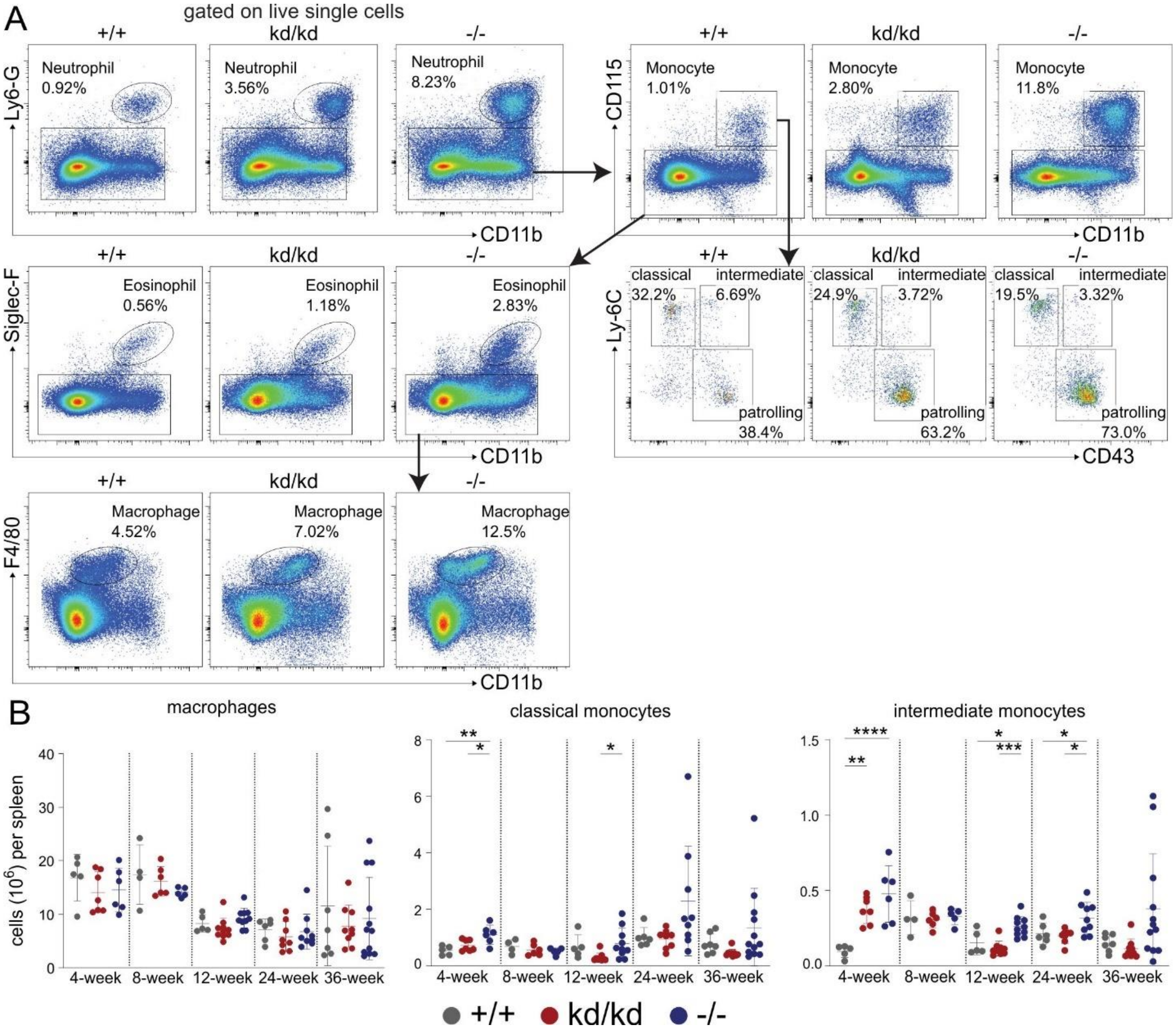

Fig. S7. Spleen myeloid cell characterization. (**A**) Flow cytometry gating strategy and representative plots from 36-week-old mice. (**B**) Splenic macrophages and monocyte subsets enumerated by flow cytometry from mice at the indicated ages, data compiled from eight independent experiments (n = 5 to 7 Lyn$^{+/+}$, 6 to 9 Lyn$^{kd/kd}$, 5 to 12 Lyn$^{-/-}$ mice per timepoint). Horizontal bars indicate mean ± SD. **P* < 0.05, ***P* < 0.01, ****P* < 0.001, *****P* < 0.0001 by one-way ANOVA with Holm-Šídák's multiple comparisons test.

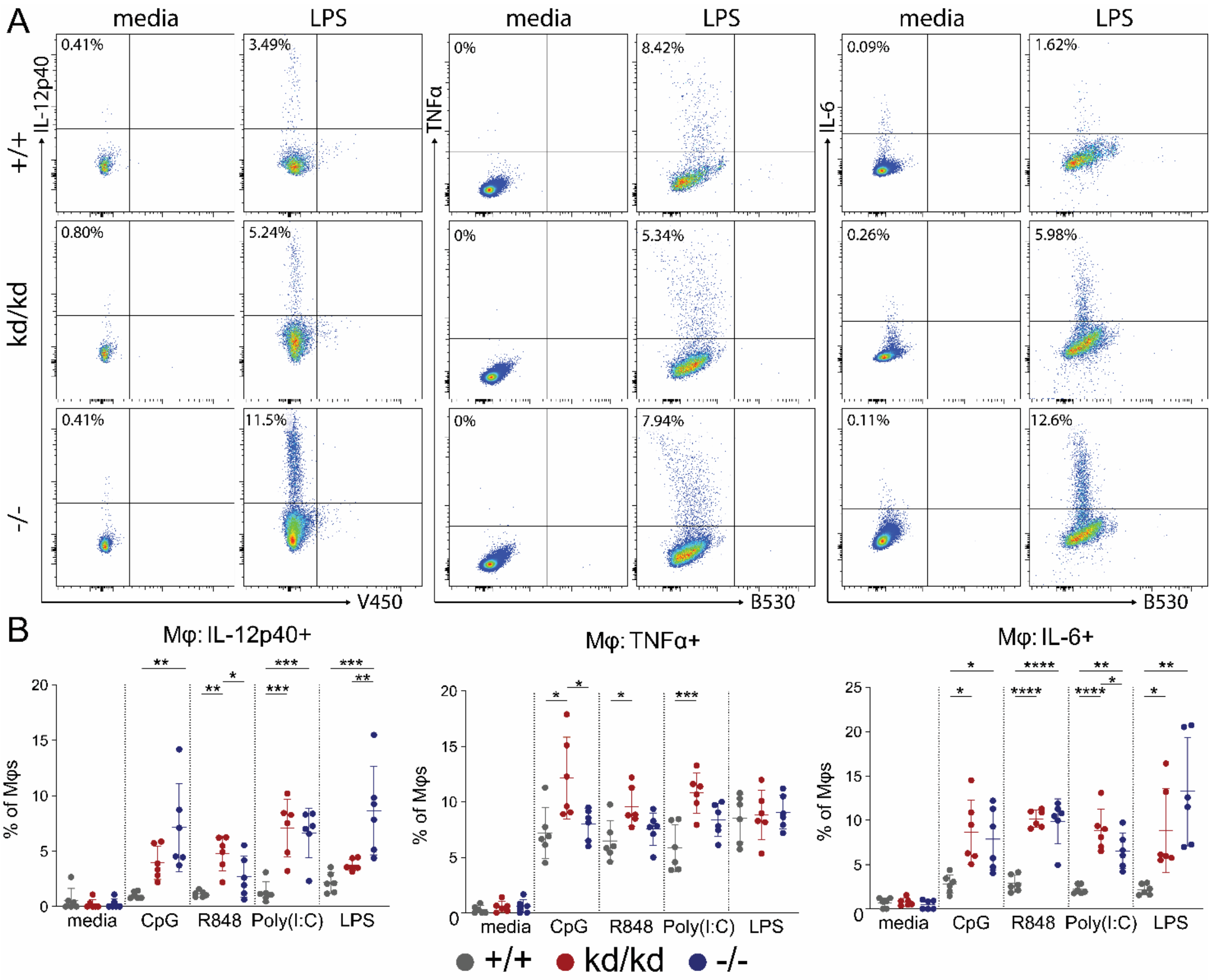

Fig. S8. Lyn$^{kd/kd}$ and Lyn$^{-/-}$ splenic macrophages from 4-6-week-old mice show similar cytokine production to TLR stimulation. (**A**) Representative plots of splenic macrophages showing intracellular cytokine staining on the vertical axes and empty channels (B530 or V450) on the horizontal axes. Staining controls were stimulated samples stained with isotype control antibodies, and anti-cytokine staining of unstimulated samples. (**B**) Frequencies of cytokine positive splenic macrophages (Mφs) showing, splenocytes stimulated for 4 hours with 1 μM CpG-C ODN 2395, 5 μg/mL R848, 5 μg/mL Poly (I:C), 5 μg/mL LPS or media in the presence of BD GolgiPlug (n = 6 per genotype; 4-6-week-old mice), data representative of two independent experiments. Horizontal bars indicate mean ± SD. $*P < 0.05$, $**P < 0.01$, $***P < 0.001$, $****P < 0.0001$ by one-way ANOVA with Holm-Šídák's multiple comparisons test.

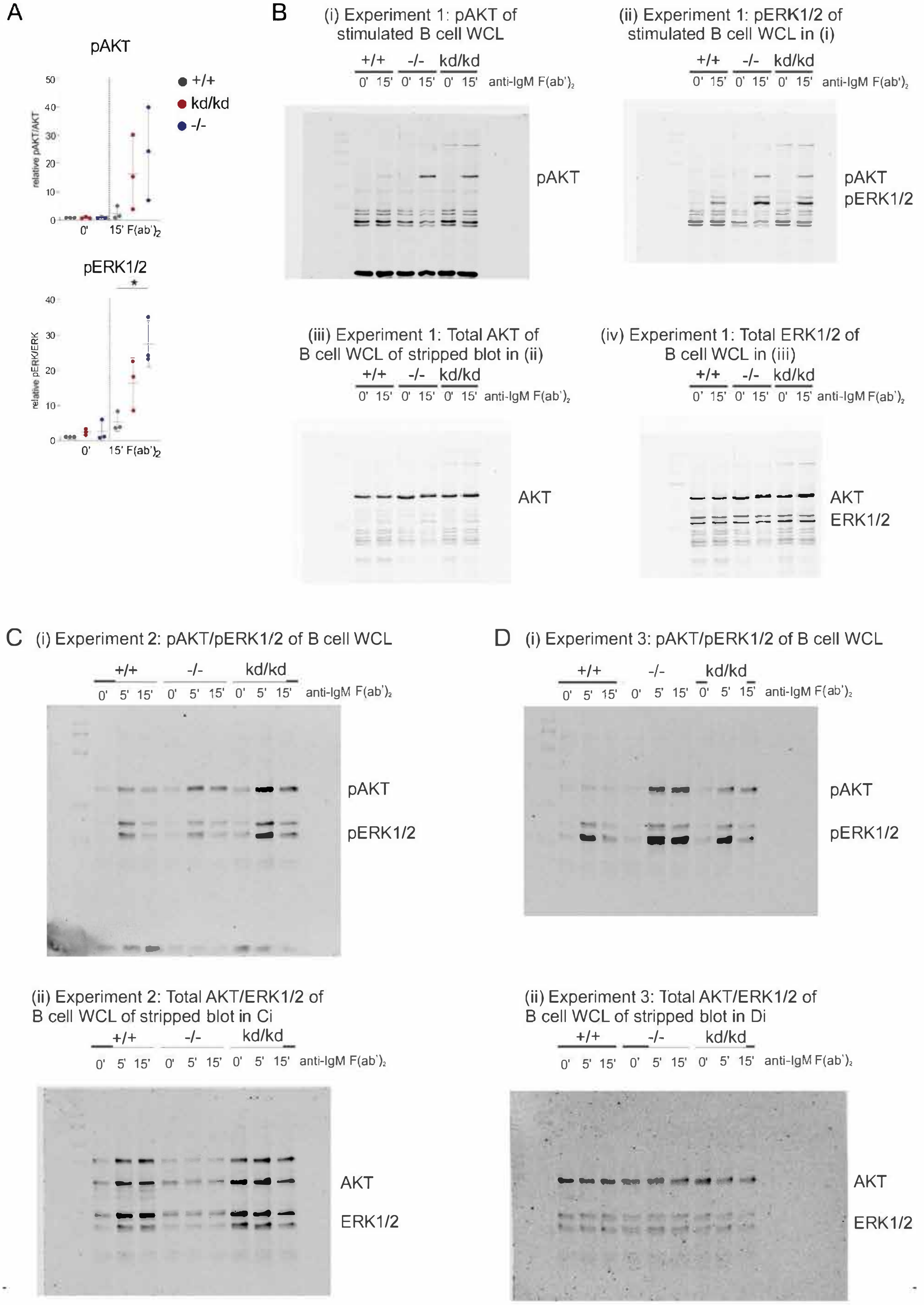
A
pAKT
+/+
kd/kd
-/-
relative pAKT/AKT
0'
15' F(ab')2
pERK1/2
relative pERK/ERK
B
(i) Experiment 1: pAKT of stimulated B cell WCL
+/+ -/- kd/kd
0' 15' 0' 15' 0' 15' anti-IgM F(ab')2
pAKT
(ii) Experiment 1: pERK1/2 of stimulated B cell WCL in (i)
pAKT
pERK1/2
(iii) Experiment 1: Total AKT of B cell WCL of stripped blot in (ii)
AKT
(iv) Experiment 1: Total ERK1/2 of B cell WCL in (iii)
AKT
ERK1/2
C
(i) Experiment 2: pAKT/pERK1/2 of B cell WCL
+/+ -/- kd/kd
0' 5' 15' 0' 5' 15' 0' 5' 15' anti-IgM F(ab')2
pAKT
pERK1/2
(ii) Experiment 2: Total AKT/ERK1/2 of B cell WCL of stripped blot in Ci
AKT
ERK1/2
D
(i) Experiment 3: pAKT/pERK1/2 of B cell WCL
pAKT
pERK1/2
(ii) Experiment 3: Total AKT/ERK1/2 of B cell WCL of stripped blot in Di
AKT
ERK1/2

Fig. S9. Biochemical characterization of B lymphocytes from Lyn$^{-/-}$ and Lyn$^{kd/kd}$ mice. (**A**) Densitometry of phospho-AKT (pAKT) and phospho-ERK1/2 (pERK1/2) from purified splenic B cells unstimulated or stimulated for 15 min with 20 μg/mL F(ab')$_2$ anti-IgM (n = 3 mice/genotype), data compiled from three independent biochemical experiments (B to D); representative cropped immunoblot shown in **Fig. 4B**. (**B**) Uncropped Western blots of the data shown in **Fig. 4B** and quantitated in (A). Blot was first probed with (i) anti-pAKT and then (ii) anti-pERK1/2, followed by stripping and reprobing with (iii) anti-AKT then (iv) anti-ERK1/2. (**C**, **D**) Uncropped Western blots from two additional experiments of purified splenic B cells from Lyn$^{+/+}$, Lyn$^{-/-}$, and Lyn$^{kd/kd}$ mice stimulated with anti-IgM F(ab')$_2$ for 0, 5, and 15 min. Blots were probed with (i) anti-pAKT and anti-pERK1/2, stripped and then reprobed with (ii) anti-AKT and anti-ERK1/2. The data from the 0 and 15 min timepoints quantitated in (A).

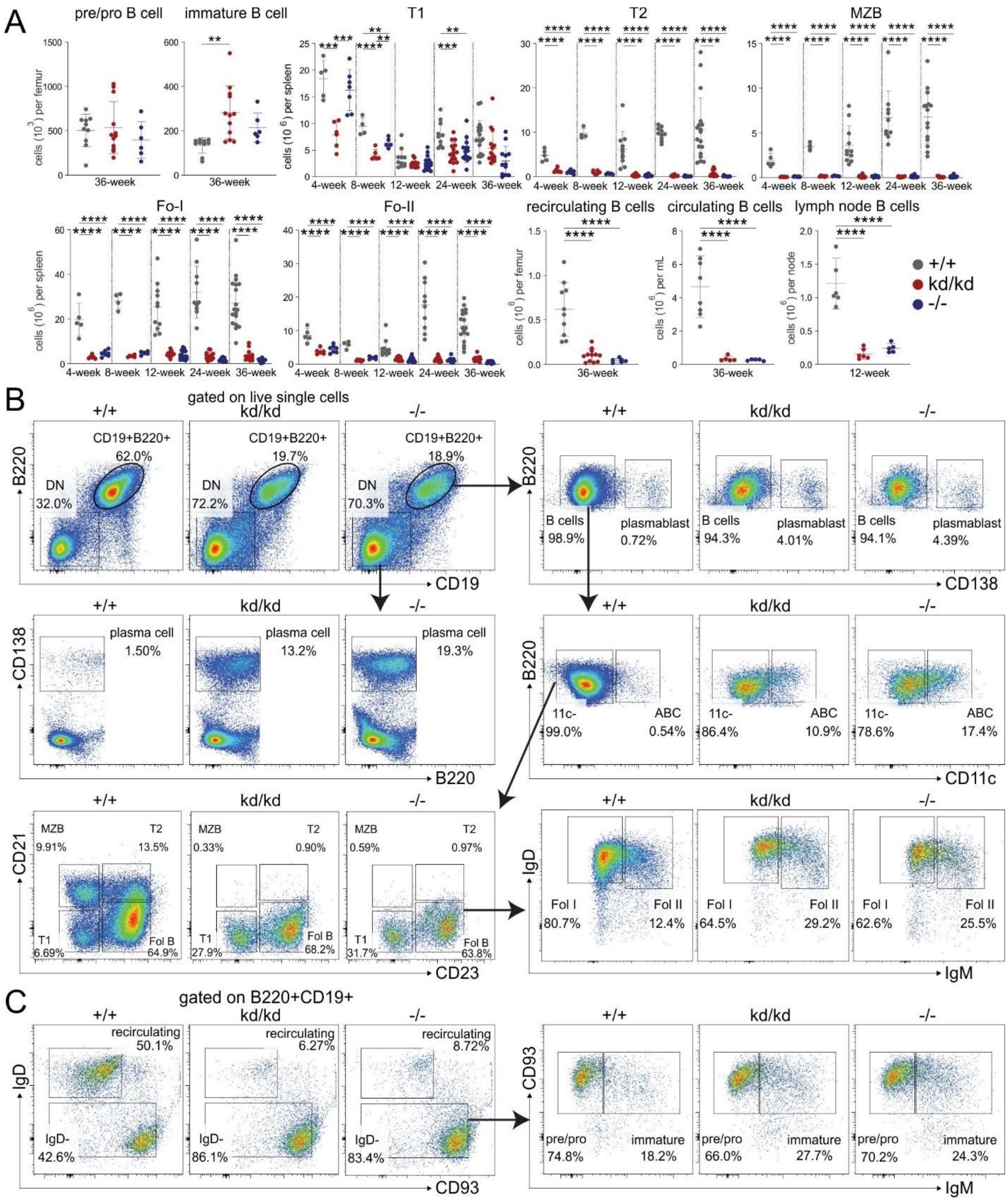

Fig. S10. Characterization of B lymphocytes in Lyn$^{-/-}$ and Lyn$^{kd/kd}$ mice. (**A**) B lymphocyte numbers in BM, spleen, blood and brachial lymph node from mice at the indicated ages (n = 4 to 18 Lyn$^{+/+}$, 6 to 17 Lyn$^{kd/kd}$, 5 to 18 Lyn$^{-/-}$ mice per timepoint), data compiled from 12 independent experiments. Horizontal bars indicate mean ± SD. **P* < 0.05, ***P* < 0.01, ****P* < 0.001, *****P* < 0.0001 by one-way ANOVA with Holm-Šídák's multiple comparisons. (**B**) Flow cytometry gating strategy for splenic B lymphocyte subsets and (**C**) BM B lymphocyte subsets, representative plots in C and D from 36-week-old mice.

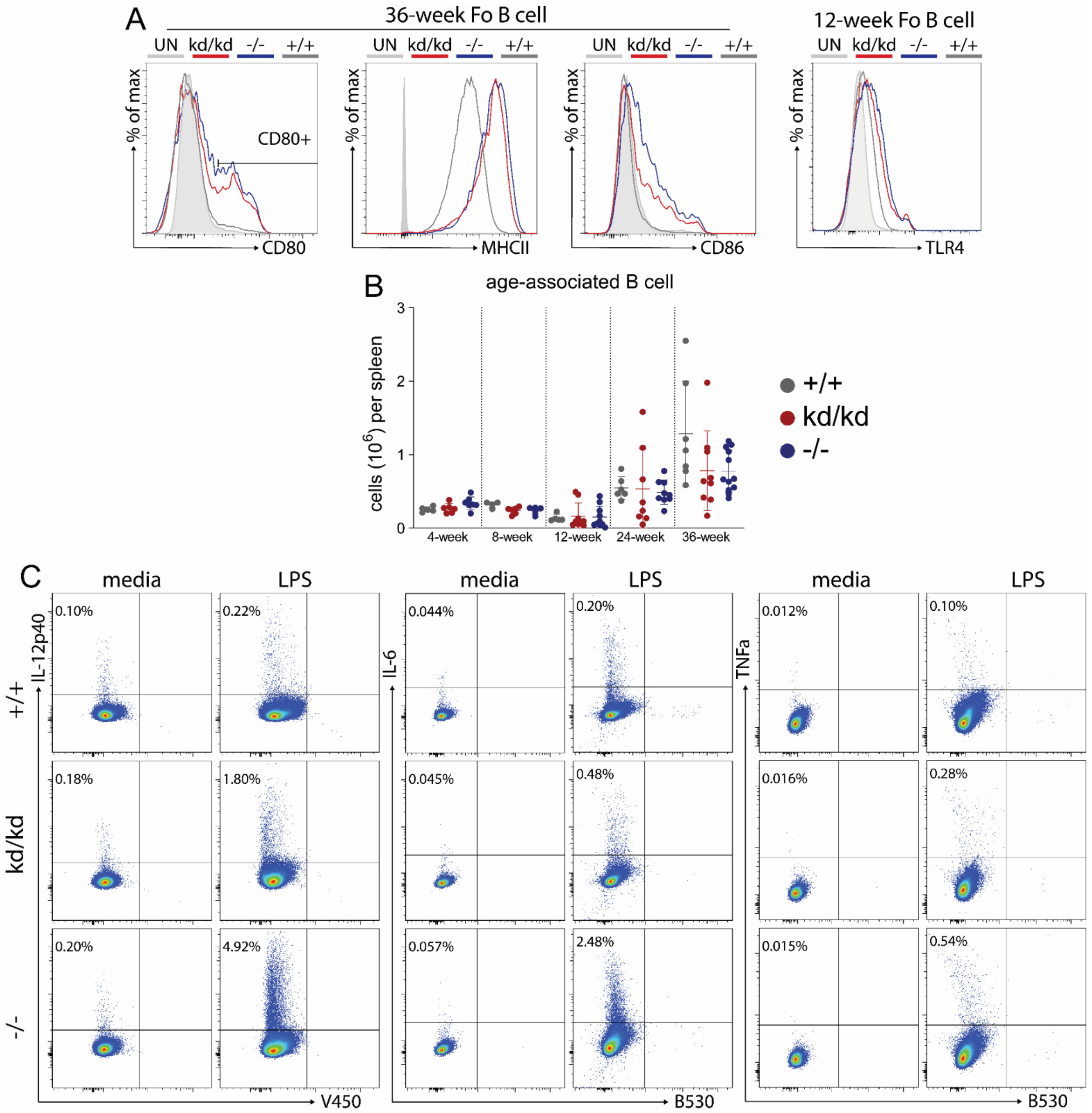

Fig. S11. B lymphocyte costimulatory marker expression and cytokine production. (**A**) Representative histograms of splenic Fo B cell activation marker expression from 36-week-old mice, unstained sample; UN is represented by the filled histogram. (**B**) Splenic age-associated B cells enumerated by flow cytometry at the indicated ages (n = 4 to 7 Lyn$^{+/+}$, 6 to 9 Lyn$^{kd/kd}$, 5 to 12 Lyn$^{-/-}$ mice per timepoint), data compiled from eight independent experiments. (**C**) Representative plots of B lymphocyte intracellular cytokine staining of samples cultured with media or LPS in the presence of GolgiPlug for 4 hours.

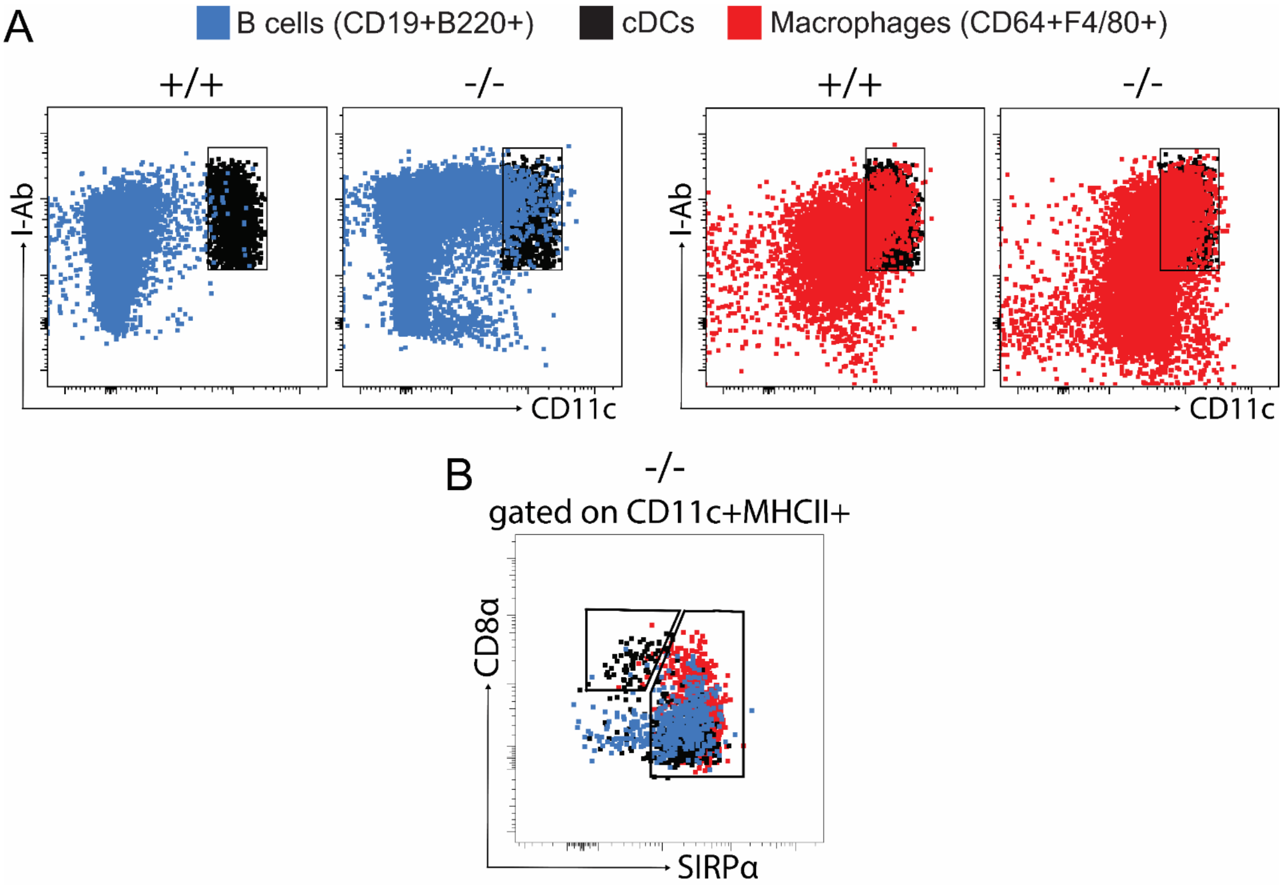

Fig. S12. ABCs and macrophages confound cDC identification in Lyn-deficient mice. (**A**) Overlay of splenic CD19+B220+ B cells (blue), CD64+F4/80+ macrophages (red), and cDCs (black, CD11c+I-Ab+B220-Siglec-H-F4/80-CD64-). (**B**) Plot of CD11c+I-Ab+ cells from Lyn$^{-/-}$ mice showing that ABCs and macrophages primarily contaminate the SIRPα+ cDC2 gate. Representative plots from 36-week-old mice.

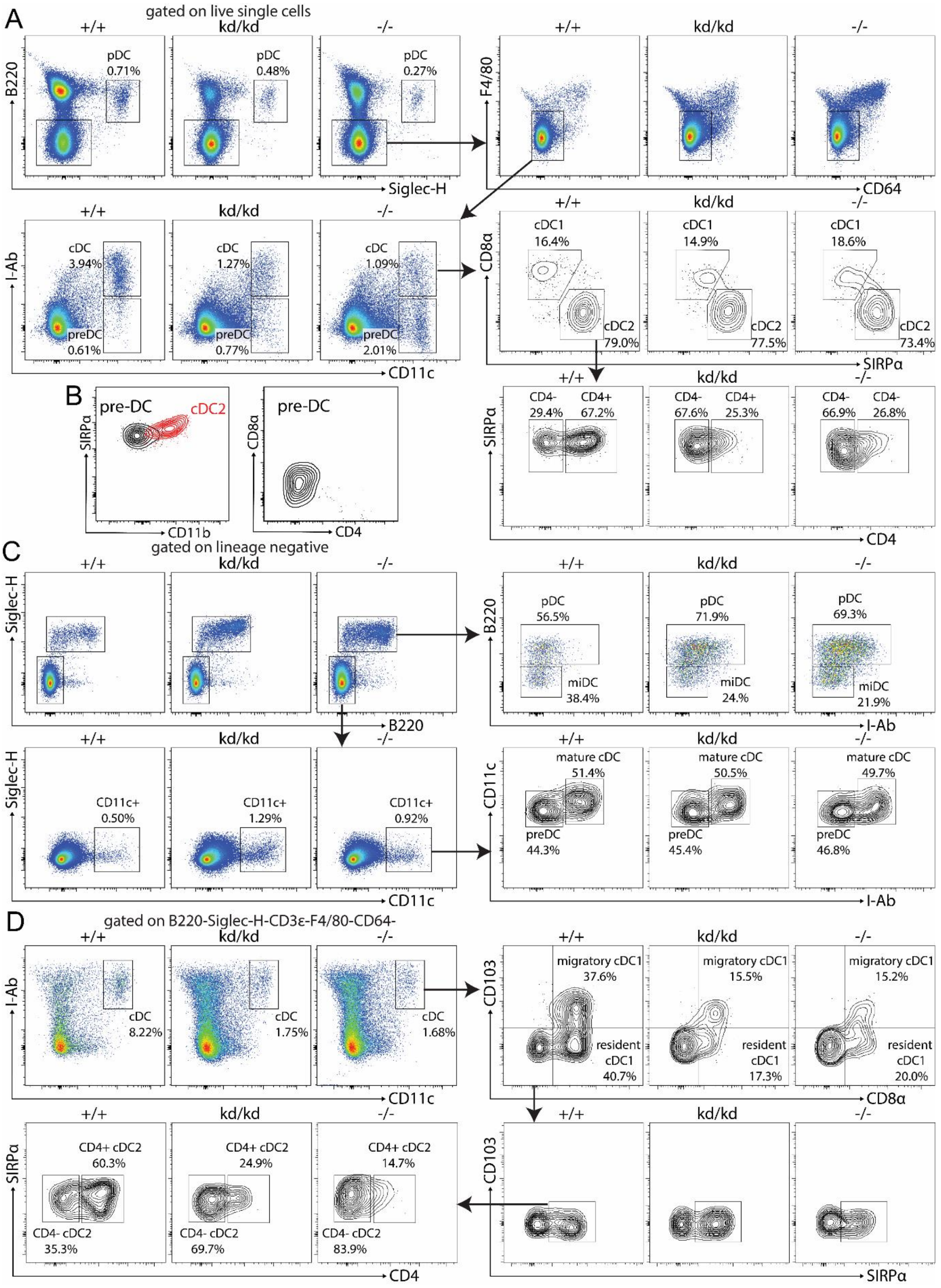
A
gated on live single cells
+/+
kd/kd
-/-
B220
Siglec-H
pDC 0.71%
pDC 0.48%
pDC 0.27%
F4/80
CD64
I-Ab
CD11c
cDC 3.94%
preDC 0.61%
cDC 1.27%
preDC 0.77%
cDC 1.09%
preDC 2.01%
CD8α
SIRPα
cDC1 16.4%
cDC2 79.0%
cDC1 14.9%
cDC2 77.5%
cDC1 18.6%
cDC2 73.4%
CD4
CD4- 29.4%
CD4+ 67.2%
CD4- 67.6%
CD4+ 25.3%
CD4- 66.9%
CD4- 26.8%
B
pre-DC
cDC2
CD11b
C
gated on lineage negative
pDC 56.5%
miDC 38.4%
pDC 71.9%
miDC 24.%
pDC 69.3%
miDC 21.9%
CD11c+ 0.50%
CD11c+ 1.29%
CD11c+ 0.92%
mature cDC 51.4%
preDC 44.3%
mature cDC 50.5%
preDC 45.4%
mature cDC 49.7%
preDC 46.8%
D
gated on B220-Siglec-H-CD3ε-F4/80-CD64-
cDC 8.22%
cDC 1.75%
cDC 1.68%
CD103
migratory cDC1 37.6%
resident cDC1 40.7%
migratory cDC1 15.5%
resident cDC1 17.3%
migratory cDC1 15.2%
resident cDC1 20.0%
CD4+ cDC2 60.3%
CD4- cDC2 35.3%
CD4+ cDC2 24.9%
CD4- cDC2 69.7%
CD4+ cDC2 14.7%
CD4- cDC2 83.9%

Fig. S13. Characterization of the DC compartment in $Lyn^{-/-}$ and $Lyn^{kd/kd}$ mice. (**A**) Flow cytometry gating strategy for splenic DC subsets, representative plots from 36-week-old mice. (**B**) Left: overlay of $Lyn^{-/-}$ splenic putative pre-DC (black) and cDC2 (red) populations, showing that the pre-DC population is SIRPα-positive and CD11b-negative, in contrast to cDC2s that are double-positive. Right: plot of $Lyn^{-/-}$ pre-DCs showing they are negative for CD8α and CD4 expression, consistent with an intrasplenic pre-cDC population (*43*). (**C**) Flow cytometry gating strategy for BM DC subsets (lineage negative: SiglecF-CD115-Ly6G-Ter119-CD4-CD8α-NK1.1-CD19-) and (**D**) brachial lymph node subsets, representative plots from 12-week-old mice.

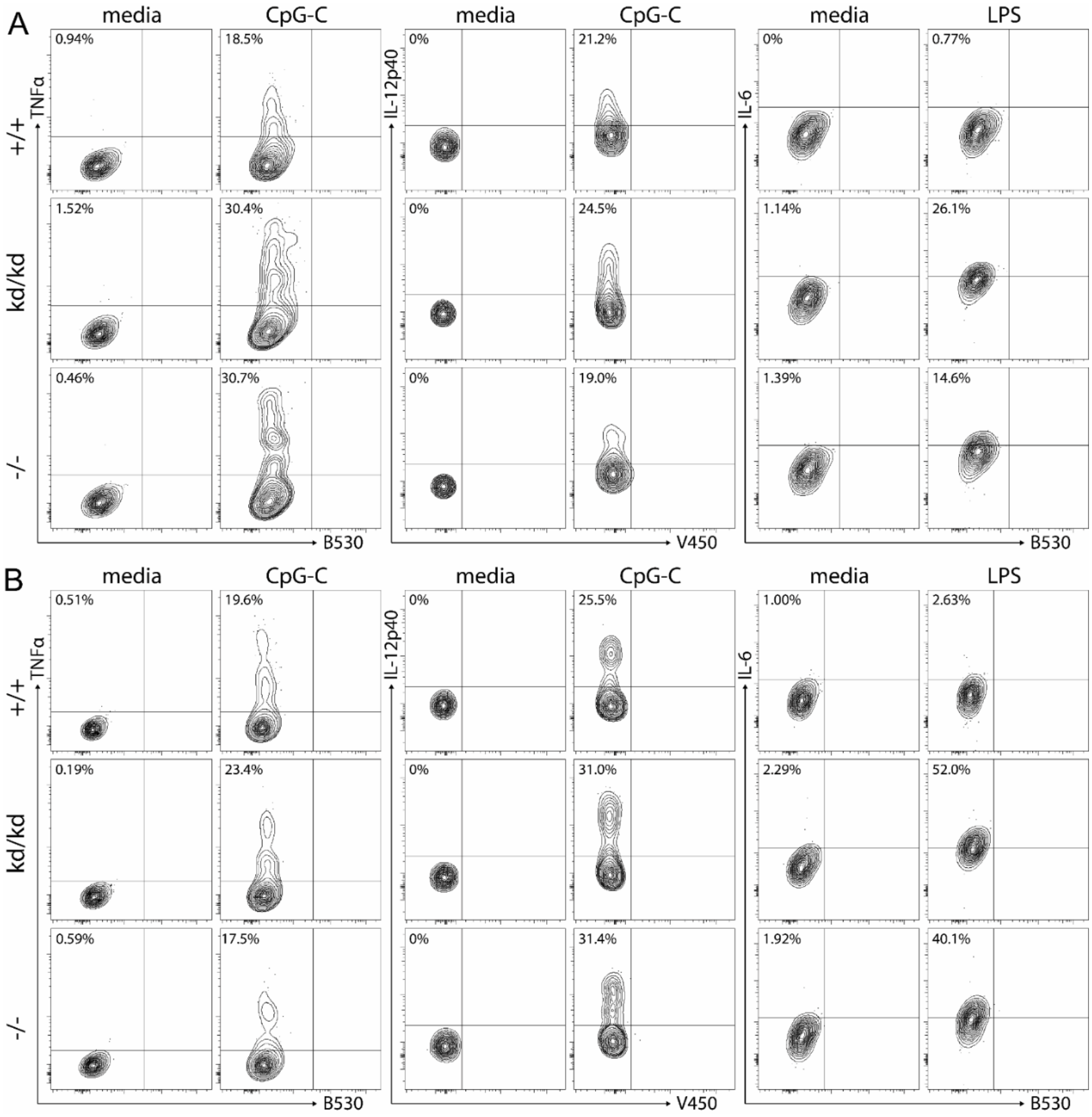

Fig. S14. Splenic cDC intracellular cytokine staining. Representative plots of (**A**) cDC1 and (**B**) cDC2 intracellular cytokine staining showing intracellular TNFα, IL-12p40, and IL-6 staining on the vertical axes and empty channels (B530 or V450) on the horizontal axes, from samples cultured with media, CpG-C, or LPS in the presence of GolgiPlug for 4 hours. Staining controls were stimulated samples stained with isotype control antibodies, and anti-cytokine staining of unstimulated samples.

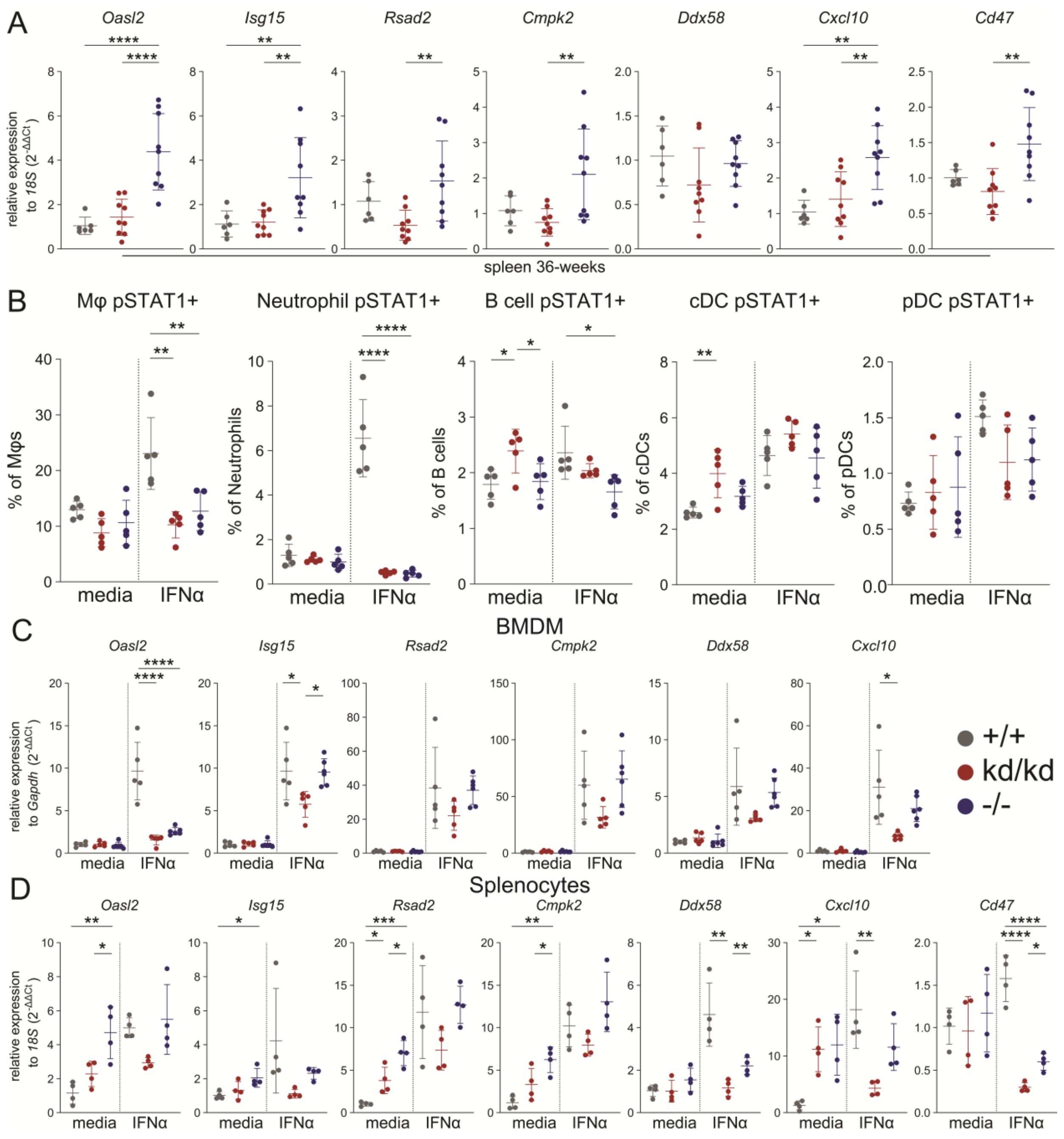

Fig. S15. Kinase-dead Lyn limits IFN-I responsiveness. (**A**) ISG expression in spleen tissue from 36-week-old (n = 6 Lyn$^{+/+}$, 9 Lyn$^{kd/kd}$, 9 Lyn$^{-/-}$) mice determined by RT-PCR, used to calculate average ISG expression shown in **Fig. 6F**. (**B**) Phospho-STAT1 quantification of data shown in **Fig. 6G** (n = 5 mice per genotype). (**C**) BMDM and (**D**) splenocyte ISG expression determined by RT-PCR in cells cultured with media or 1000 IU/mL IFNα for 8 hours (n = 4-5 Lyn$^{+/+}$, 4-5 Lyn$^{kd/kd}$, 4-6 Lyn$^{-/-}$ mice), used to calculate average ISG expression shown in **Fig. 6H**. Horizontal bars indicate mean ± SD. **P* < 0.05, ***P* < 0.01, ****P* < 0.001, *****P* < 0.0001 by one-way ANOVA with Holm-Šídák's multiple comparisons test.

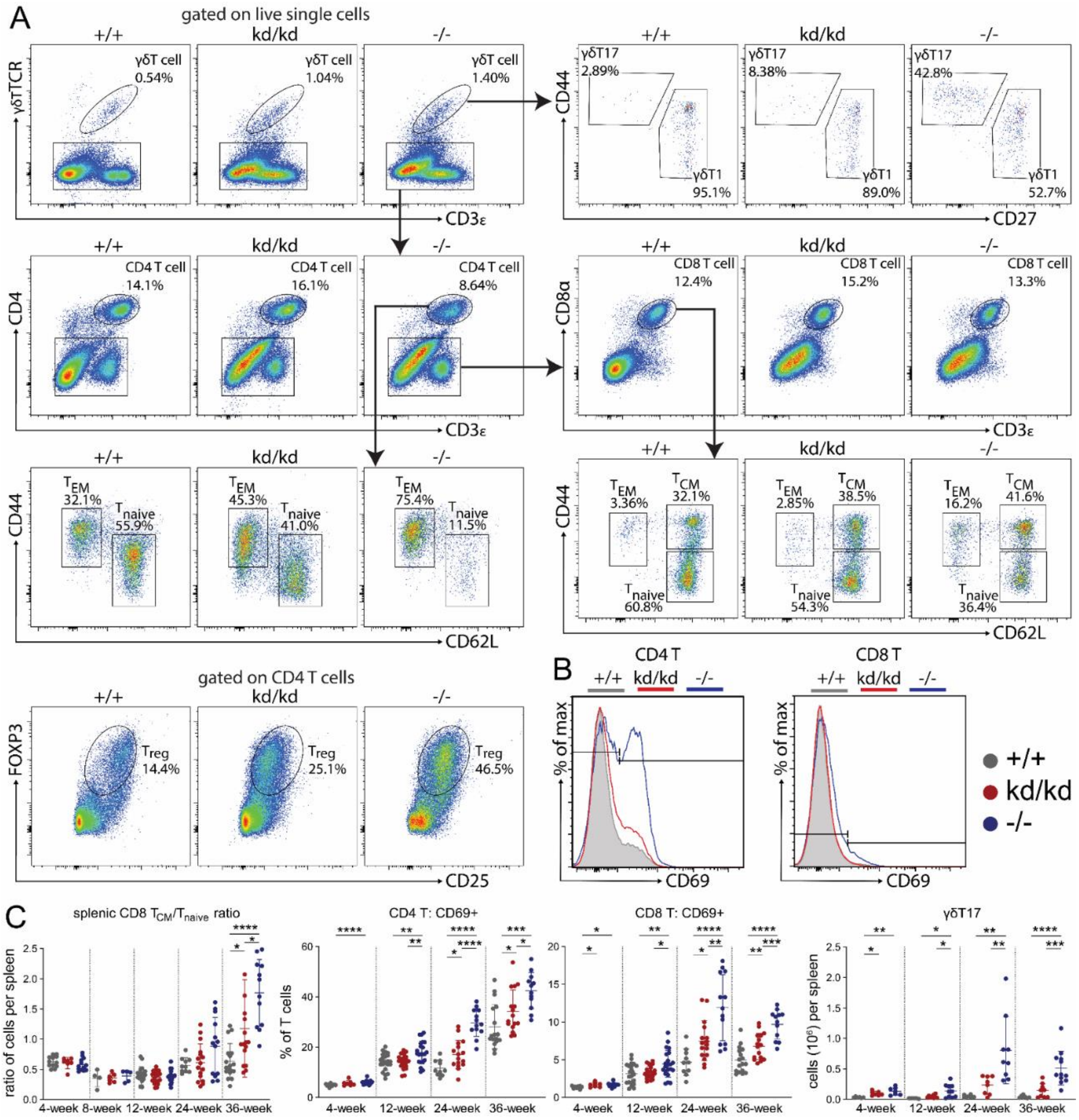

Fig. S16. Splenic T cell flow cytometry profiling. (**A**) Gating strategy and representative plots from 36-week-old mice. (**B**) Representative histograms of CD69 expression of CD4 T and CD8 T cells from 36-week-old mice. (**C**) Left: ratio of central memory CD8 T cells to naïve CD8 T cells, middle: percentage of CD69+ CD4 and CD8 T cells, and right: numbers of γδT17 cells, enumerated by flow cytometry of splenocytes from mice at the indicated ages, data compiled from 14 independent experiments (n = 4 to 21 $Lyn^{+/+}$, 4 to 20 $Lyn^{kd/kd}$, 5 to 22 $Lyn^{-/-}$ mice per timepoint). Horizontal bars indicate mean ± SD. **P* < 0.05, ***P* < 0.01, ****P* < 0.001, *****P* < 0.0001 by one-way ANOVA with Holm-Šídák's multiple comparisons test.

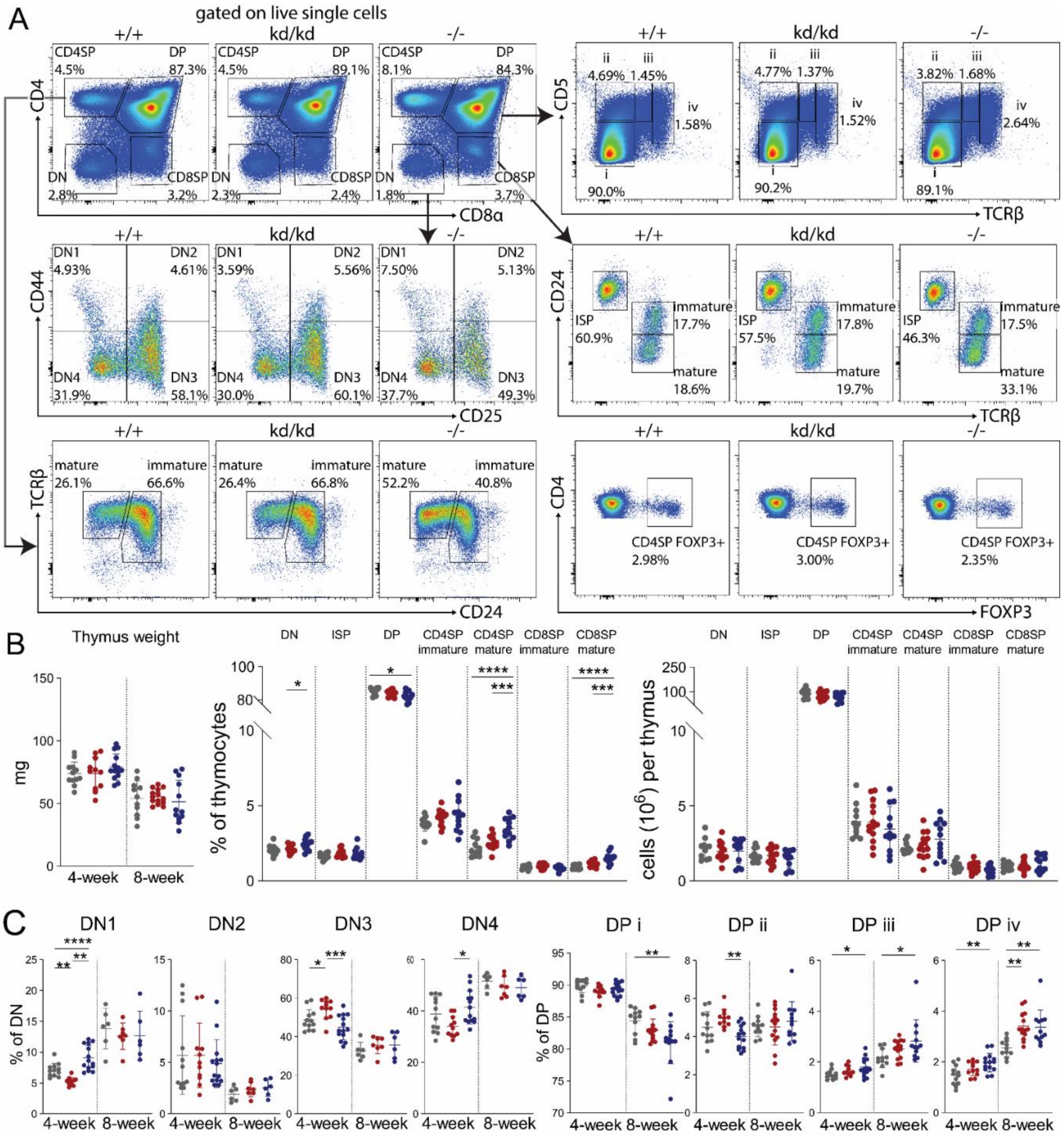

Fig. S17. Thymocyte profiling. (**A**) Thymocyte gating strategy from 4-week-old mice and FOXP3 staining of CD4SP+ thymocytes from 8-week-old mice. (**B**) Left: thymus weights, and right: thymocyte frequencies and numbers from 8-week-old mice. (**C**) Double-Negative (DN) and Double-Positive (DP) subset frequencies. (B and C) Data compiled from four independent experiments (n = 6 to 12 Lyn$^{+/+}$, 7 to 14 Lyn$^{kd/kd}$, 6 to 14 Lyn$^{-/-}$ mice per timepoint). Horizontal bars indicate mean ± SD. *P < 0.05, **P < 0.01, ***P < 0.001, ****P < 0.0001 by one-way ANOVA with Holm-Šídák's multiple comparisons test.

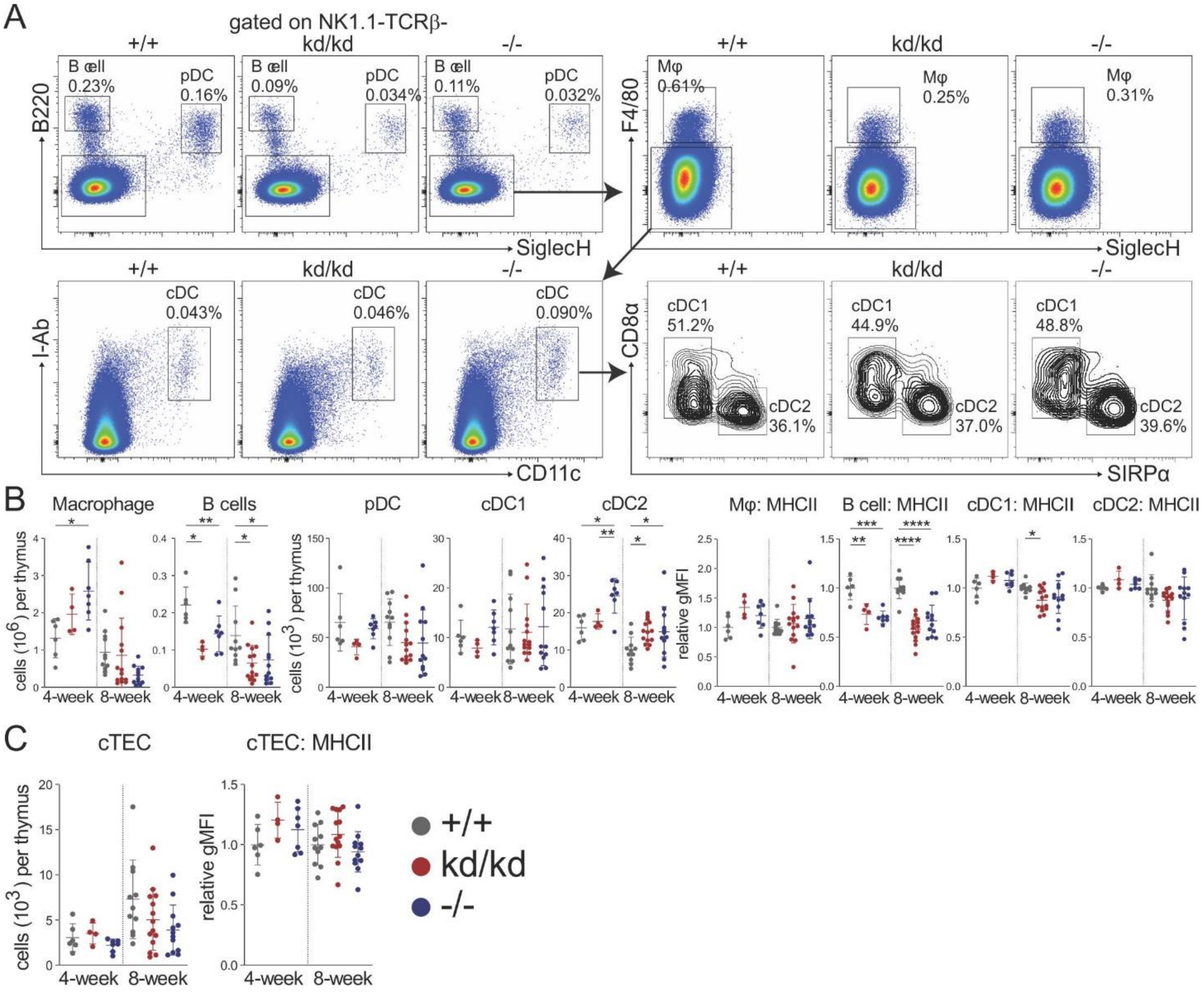

Fig. S18. Thymic APC profiling. (**A**) Thymic APC gating strategy and representative plots from 8-week-old-mice. (**B**) Left: Thymic APC numbers and right: MHCII expression (relative gMFI). (**C**) Cortical thymic epithelial cell numbers and MHCII expression (relative gMFI), based on thymic epithelial cell gating strategy shown in Figure 7C. (B and C) Data compiled from three independent experiments (n = 6 to 11 $Lyn^{+/+}$, 4 to 14 $Lyn^{kd/kd}$, 7 to 12 $Lyn^{-/-}$ mice per timepoint). Horizontal bars indicate mean ± SD. **P* < 0.05, ***P* < 0.01, ****P* < 0.001, *****P* < 0.0001 by one-way ANOVA with Holm-Šídák's multiple comparisons test.

**Gene symbol**	**Gene name**	**Reference**
Acta2	Smooth muscle alpha-2 actin	In-house
Baff	B cell-activating factor of the tumour necrosis factor family	(*4*)
Ccl2	C-C motif chemokine ligand 2	(*84*)
Cd47	Integrin-associated protein	In-house
Cmpk2	Cytidine/Uridine Monophosphate Kinase 2	In-house
Col1a1	Collagen Type I Alpha 1	(*85*)
Cxcl1	CXC motif chemokine ligand 1	(*86*)
Cxcl10	CXC motif chemokine ligand 10	In-house
Cxcl11	CXC motif chemokine ligand 11	In-house
Cxcl9	CXC motif chemokine ligand 9	In-house
Ddx58	DExD/H-box helicase 58	(*72*)
Fgg	Fibrinogen gamma	(*85*)
Grzb	Granzyme B	In-house
Ifna	Interferon alpha	(*22*)
Ifnb1	Interferon beta 1	(*22*)
Ifng	Interferon gamma	In-house
Il12b	Interleukin 12b	In-house
Il17a	Interleukin 17a	*(69)*
Il1b	Interleukin 1b	*(87)*
Il6	Interleukin 6	*(69)*
Irf7	Interferon regulatory factor 7	In-house
Isg15	Interferon-stimulated gene 15	In-house
Kim1	Kidney injury molecule 1	*(88)*
Lyn	Lyn tyrosine kinase	In-house
Mmp12	Matrix metalloproteinase 12	*(89)*
Nox2	NADPH oxidase 2	*(90)*
Oasl2	2’-5’Oligoadenylate synthetase-like 2	In-house
Rsad2	Radical S-adenosyl methionine domain containing 2	In-house
Tnfa	Tumour necrosis factor alpha	*(8)*
18S	18S ribosomal RNA	*(91)*
Gapdh	Glyceraldehyde-3-phosphate dehydrogenase	*(92)*

Table S1. List of primers used for RT-PCR and the source reference. The specific nucleotide sequences of the forward and reverse primers used to amplify each gene are presented in Supplementary File S2.

The following supplementary files are contained in adz1726_Suppl. Excel_seq1_V1.xlsx

Supplementary File S1. Commercial antibodies

Supplementary File S2. qPCR primer sequences

REFERENCES AND NOTES

1. T. A. Gottschalk, E. Tsantikos, M. L. Hibbs, Pathogenic inflammation and its therapeutic targeting in systemic lupus erythematosus. *Front. Immunol.* **6**, 550 (2015).

2. C. Lamagna, Y. Hu, A. L. DeFranco, C. A. Lowell, B cell-specific loss of Lyn kinase leads to autoimmunity. *J. Immunol.* **192**, 919–928 (2014).

3. C. Lamagna, P. Scapini, J. A. van Ziffle, A. L. DeFranco, C. A. Lowell, Hyperactivated MyD88 signaling in dendritic cells, through specific deletion of Lyn kinase, causes severe autoimmunity and inflammation. *Proc. Natl. Acad. Sci. U.S.A.* **110**, E3311–E3320 (2013).

4. P. Scapini, Y. Hu, C. L. Chu, T. S. Migone, A. L. Defranco, M. A. Cassatella, C. A. Lowell, Myeloid cells, BAFF, and IFN-γ establish an inflammatory loop that exacerbates autoimmunity in Lyn-deficient mice. *J. Exp. Med.* **207**, 1757–1773 (2010).

5. M. L. Hibbs, D. M. Tarlinton, J. Armes, D. Grail, G. Hodgson, R. Maglitto, S. A. Stacker, A. R. Dunn, Multiple defects in the immune system of Lyn-deficient mice, culminating in autoimmune disease. *Cell* **83**, 301–311 (1995).

6. H. Nishizumi, I. Taniuchi, Y. Yamanashi, D. Kitamura, D. Ilic, S. Mori, T. Watanabe, T. Yamamoto, Impaired proliferation of peripheral B cells and indication of autoimmune disease in lyn-deficient mice. *Immunity* **3**, 549–560 (1995).

7. E. Tsantikos, S. A. Oracki, C. Quilici, G. P. Anderson, D. M. Tarlinton, M. L. Hibbs, Autoimmune disease in Lyn-deficient mice is dependent on an inflammatory environment established by IL-6. *J. Immunol.* **184**, 1348–1360 (2010).

8. T. A. Gottschalk, P. Hall, E. Tsantikos, E. L'Estrange-Stranieri, M. J. Hickey, M. L. Hibbs, Loss of CD11b accelerates lupus nephritis in Lyn-deficient mice without disrupting glomerular leukocyte trafficking. *Front. Immunol.* **13**, 875359 (2022).

9. E. L'Estrange-Stranieri, T. A. Gottschalk, M. D. Wright, M. L. Hibbs, The dualistic role of Lyn tyrosine kinase in immune cell signaling: Implications for systemic lupus erythematosus. *Front. Immunol.* **15**, 1395427 (2024).

10. K. W. Harder, L. M. Parsons, J. Armes, N. Evans, N. Kountouri, R. Clark, C. Quilici, D. Grail, G. S. Hodgson, A. R. Dunn, M. L. Hibbs, Gain- and loss-of-function Lyn mutant mice define a critical inhibitory role for Lyn in the myeloid lineage. *Immunity* **15**, 603–615 (2001).

11. V. W. Chan, F. Meng, P. Soriano, A. L. DeFranco, C. A. Lowell, Characterization of the B lymphocyte populations in Lyn-deficient mice and the role of Lyn in signal initiation and down-regulation. *Immunity* **7**, 69–81 (1997).

12. H. Nishizumi, K. Horikawa, I. Mlinaric-Rascan, T. Yamamoto, A double-edged kinase Lyn: A positive and negative regulator for antigen receptor-mediated signals. *J. Exp. Med.* **187**, 1343–1348 (1998).

13. R. J. Cornall, J. G. Cyster, M. L. Hibbs, A. R. Dunn, K. L. Otipoby, E. A. Clark, C. C. Goodnow, Polygenic autoimmune traits: Lyn, CD22, and SHP-1 are limiting elements of a biochemical pathway regulating BCR signaling and selection. *Immunity* **8**, 497–508 (1998).

14. J. Jellusova, U. Wellmann, K. Amann, T. H. Winkler, L. Nitschke, CD22 x Siglec-G double-deficient mice have massively increased B1 cell numbers and develop systemic autoimmunity. *J. Immunol.* **184**, 3618–3627 (2010).

15. S. Bolland, J. V. Ravetch, Spontaneous autoimmune disease in FcγRIIB-deficient mice results from strain-specific epistasis. *Immunity* **13**, 277–285 (2000).

16. T. Kubo, Y. Uchida, Y. Watanabe, M. Abe, A. Nakamura, M. Ono, S. Akira, T. Takai, Augmented TLR9-induced Btk activation in PIR-B-deficient B-1 cells provokes excessive autoantibody production and autoimmunity. *J. Exp. Med.* **206**, 1971–1982 (2009).

17. L. I. Pao, K. P. Lam, J. M. Henderson, J. L. Kutok, M. Alimzhanov, L. Nitschke, M. L. Thomas, B. G. Neel, K. Rajewsky, B cell-specific deletion of protein-tyrosine phosphatase Shp1 promotes B-1a cell development and causes systemic autoimmunity. *Immunity* **27**, 35–48 (2007).

18. M. J. Maxwell, M. Duan, J. E. Armes, G. P. Anderson, D. M. Tarlinton, M. L. Hibbs, Genetic segregation of inflammatory lung disease and autoimmune disease severity in SHIP-1$^{-/-}$ mice. *J. Immunol.* **186**, 7164–7175 (2011).

19. J. Rauch, N. Volinsky, D. Romano, W. Kolch, The secret life of kinases: Functions beyond catalysis. *Cell Commun. Signal* **9**, 23 (2011).

20. H. Katsuta, S. Tsuji, Y. Niho, T. Kurosaki, D. Kitamura, Lyn-mediated down-regulation of B cell antigen receptor signaling: Inhibition of protein kinase C activation by Lyn in a kinase-independent fashion. *J. Immunol.* **160**, 1547–1551 (1998).

21. X. Ren, C. Cao, L. Zhu, K. Yoshida, S. Kharbanda, R. Weichselbaum, D. Kufe, Lyn tyrosine kinase inhibits nuclear export of the p53 tumor suppressor. *Cancer Biol. Ther.* **1**, 703–708 (2002).

22. T. Ban, G. R. Sato, A. Nishiyama, A. Akiyama, M. Takasuna, M. Umehara, S. Suzuki, M. Ichino, S. Matsunaga, A. Kimura, Y. Kimura, H. Yanai, S. Miyashita, J. Kuromitsu, K. Tsukahara, K. Yoshimatsu, I. Endo, T. Yamamoto, H. Hirano, A. Ryo, T. Taniguchi, T. Tamura, Lyn kinase suppresses the transcriptional activity of IRF5 in the TLR-MyD88 pathway to restrain the development of autoimmunity. *Immunity* **45**, 319–332 (2016).

23. K. L. Silver, T. L. Crockford, T. Bouriez-Jones, S. Milling, T. Lambe, R. J. Cornall, MyD88-dependent autoimmune disease in Lyn-deficient mice. *Eur. J. Immunol.* **37**, 2734–2743 (2007).

24. Z. Hua, A. J. Gross, C. Lamagna, N. Ramos-Hernandez, P. Scapini, M. Ji, H. Shao, C. A. Lowell, B. Hou, A. L. DeFranco, Requirement for MyD88 signaling in B cells and dendritic cells for germinal center anti-nuclear antibody production in Lyn-deficient mice. *J. Immunol.* **192**, 875–885 (2014).

25. K. Tawaratsumida, V. Redecke, R. Wu, J. Kuriakose, J. J. Bouchard, T. Mittag, B. K. Lohman, A. Mishra, A. A. High, H. Hacker, A phospho-tyrosine-based signaling module using SPOP, CSK, and LYN controls TLR-induced IRF activity. *Sci. Adv.* **8**, eabq0084 (2022).

26. J. Ma, C. L. Abram, Y. Hu, C. A. Lowell, CARD9 mediates dendritic cell–induced development of Lyn deficiency–associated autoimmune and inflammatory diseases. *Sci. Signal.* **12**, eaao3829 (2019).

27. M. Dalod, S. Scheu, Dendritic cell functions in vivo: A user's guide to current and next-generation mutant mouse models. *Eur. J. Immunol.* **52**, 1712–1749 (2022).

28. R. Barouch-Bentov, J. Che, C. C. Lee, Y. Yang, A. Herman, Y. Jia, A. Velentza, J. Watson, L. Sternberg, S. Kim, N. Ziaee, A. Miller, C. Jackson, M. Fujimoto, M. Young, S. Batalov, Y. Liu, M. Warmuth, T. Wiltshire, M. P. Cooke, K. Sauer, A conserved salt bridge in the G loop of multiple protein kinases is important for catalysis and for in vivo Lyn function. *Mol. Cell* **33**, 43–52 (2009).

29. A. M. Verhagen, M. E. Wallace, A. Goradia, S. A. Jones, H. A. Croom, D. Metcalf, J. E. Collinge, M. J. Maxwell, M. L. Hibbs, W. S. Alexander, D. J. Hilton, B. T. Kile, R. Starr, A kinase-dead allele of Lyn attenuates autoimmune disease normally associated with Lyn deficiency. *J. Immunol.* **182**, 2020–2029 (2009).

30. T. A. Gottschalk, F. B. Vincent, A. Y. Hoi, M. L. Hibbs, Granulocyte colony-stimulating factor is not pathogenic in lupus nephritis. *Immun. Inflamm. Dis.* **9**, 758–770 (2021).

31. Y. Nozaki, K. Kinoshita, T. Yano, T. Shiga, S. Hino, K. Niki, K. Kishimoto, M. Funauchi, I. Matsumura, Estimation of kidney injury molecule-1 (Kim-1) in patients with lupus nephritis. *Lupus* **23**, 769–777 (2014).

32. K. M. Kingsmore, P. Bachali, M. D. Catalina, A. R. Daamen, S. E. Heuer, R. D. Robl, A. C. Grammer, P. E. Lipsky, Altered expression of genes controlling metabolism characterizes the tissue response to immune injury in lupus. *Sci. Rep.* **11**, 14789 (2021).

33. T. Kusaba, M. Lalli, R. Kramann, A. Kobayashi, B. D. Humphreys, Differentiated kidney epithelial cells repair injured proximal tubule. *Proc. Natl. Acad. Sci. U.S.A.* **111**, 1527–1532 (2014).

34. B. Smeets, M. L. Angelotti, P. Rizzo, H. Dijkman, E. Lazzeri, F. Mooren, L. Ballerini, E. Parente, C. Sagrinati, B. Mazzinghi, E. Ronconi, F. Becherucci, A. Benigni, E. Steenbergen, L. Lasagni, G. Remuzzi, J. Wetzels, P. Romagnani, Renal progenitor cells contribute to hyperplastic lesions of podocytopathies and crescentic glomerulonephritis. *J. Am. Soc. Nephrol.* **20**, 2593–2603 (2009).

35. A. R. Kitching, J. D. Ooi, Renal dendritic cells: The long and winding road. *J. Am. Soc. Nephrol.* **29**, 4–7 (2018).

36. S. Brahler, B. H. Zinselmeyer, S. Raju, M. Nitschke, H. Suleiman, B. T. Saunders, M. W. Johnson, A. M. C. Bohner, S. F. Viehmann, D. J. Theisen, N. M. Kretzer, C. G. Briseno, K. Zaitsev, O. Ornatsky, Q. Chang, J. A. Carrero, J. B. Kopp, M. N. Artyomov, C. Kurts, K. M. Murphy, J. H. Miner, A. S. Shaw, Opposing roles of dendritic cell subsets in experimental GN. *J. Am. Soc. Nephrol.* **29**, 138–154 (2018).

37. N. Richoz, Z. K. Tuong, K. W. Loudon, E. Patino-Martinez, J. R. Ferdinand, A. Portet, K. R. Bashant, E. Thevenon, F. Rucci, T. Hoyler, T. Junt, M. J. Kaplan, R. M. Siegel, M. R. Clatworthy, Distinct pathogenic roles for resident and monocyte-derived macrophages in lupus nephritis. *JCI Insight* **7**, e159751 (2022).

38. B. F. t. Brian, T. S. Freedman, The Src-family kinase Lyn in immunoreceptor signaling. *Endocrinology* **162**, bqab152 (2021).

39. K. W. Harder, C. Quilici, E. Naik, M. Inglese, N. Kountouri, A. Turner, K. Zlatic, D. M. Tarlinton, M. L. Hibbs, Perturbed myelo/erythropoiesis in Lyn-deficient mice is similar to that in mice lacking the inhibitory phosphatases SHP-1 and SHIP-1. *Blood* **104**, 3901–3910 (2004).

40. G. Shahaf, A. J. Gross, M. Sternberg-Simon, D. Kaplan, A. L. DeFranco, R. Mehr, Lyn deficiency affects B-cell maturation as well as survival. *Eur. J. Immunol.* **42**, 511–521 (2012).

41. A. J. Gross, I. Proekt, A. L. DeFranco, Elevated BCR signaling and decreased survival of Lyn-deficient transitional and follicular B cells. *Eur. J. Immunol.* **41**, 3645–3655 (2011).

42. M. Noviski, J. L. Mueller, A. Satterthwaite, L. A. Garrett-Sinha, F. Brombacher, J. Zikherman, IgM and IgD B cell receptors differentially respond to endogenous antigens and control B cell fate. *Elife* **7**, e3574 (2018).

43. S. H. Naik, D. Metcalf, A. van Nieuwenhuijze, I. Wicks, L. Wu, M. O'Keeffe, K. Shortman, Intrasplenic steady-state dendritic cell precursors that are distinct from monocytes. *Nat. Immunol.* **7**, 663–671 (2006).

44. N. Onai, T. Ohteki, Isolation of dendritic cell progenitor and bone marrow progenitor cells from mouse. *Methods Mol. Biol.* **1423**, 53–59 (2016).

45. A. Mildner, S. Jung, Development and function of dendritic cell subsets. *Immunity* **40**, 642–656 (2014).

46. T. Ban, M. Kikuchi, G. R. Sato, A. Manabe, N. Tagata, K. Harita, A. Nishiyama, K. Nishimura, R. Yoshimi, Y. Kirino, H. Yanai, Y. Matsumoto, S. Suzuki, H. Hihara, M. Ito, K. Tsukahara, K. Yoshimatsu, T. Yamamoto, T. Taniguchi, H. Nakajima, S. Ito, T. Tamura, Genetic and chemical inhibition of IRF5 suppresses pre-existing mouse lupus-like disease. *Nat. Commun.* **12**, 4379 (2021).

47. M. O'Keeffe, B. Fancke, M. Suter, G. Ramm, J. Clark, L. Wu, H. Hochrein, Nonplasmacytoid, high IFN-α-producing, bone marrow dendritic cells. *J. Immunol.* **188**, 3774–3783 (2012).

48. A. Schlitzer, J. Loschko, K. Mair, R. Vogelmann, L. Henkel, H. Einwachter, M. Schiemann, J. H. Niess, W. Reindl, A. Krug, Identification of $CCR9^{-}$ murine plasmacytoid DC precursors with plasticity to differentiate into conventional DCs. *Blood* **117**, 6562–6570 (2011).

49. Y. Gao, B. Majchrzak-Kita, E. N. Fish, J. L. Gommerman, Dynamic accumulation of plasmacytoid dendritic cells in lymph nodes is regulated by interferon-β. *Blood* **114**, 2623–2631 (2009).

50. E. Tsantikos, C. Quilici, K. W. Harder, B. Wang, H. J. Zhu, G. P. Anderson, D. M. Tarlinton, M. L. Hibbs, Perturbation of the CD4 T cell compartment and expansion of regulatory T cells in autoimmune-prone Lyn-deficient mice. *J. Immunol.* **183**, 2484–2494 (2009).

51. M. Hinterberger, M. Aichinger, O. Prazeres da Costa, D. Voehringer, R. Hoffmann, L. Klein, Autonomous role of medullary thymic epithelial cells in central $CD4^+$ T cell tolerance. *Nat. Immunol.* **11**, 512–519 (2010).

52. O. Herbin, A. J. Bonito, S. Jeong, E. G. Weinstein, A. H. Rahman, H. Xiong, M. Merad, K. Alexandropoulos, Medullary thymic epithelial cells and $CD8\alpha^+$ dendritic cells coordinately regulate central tolerance but $CD8\alpha^+$ cells are dispensable for thymic regulatory T cell production. *J. Autoimmun.* **75**, 141–149 (2016).

53. H. Takaba, H. Takayanagi, The mechanisms of T cell selection in the thymus. *Trends Immunol.* **38**, 805–816 (2017).

54. L. Klein, B. Kyewski, P. M. Allen, K. A. Hogquist, Positive and negative selection of the T cell repertoire: What thymocytes see (and don't see). *Nat. Rev. Immunol.* **14**, 377–391 (2014).

55. S. Endo, Y. Sakamoto, E. Kobayashi, A. Nakamura, T. Takai, Regulation of cytotoxic T lymphocyte triggering by PIR-B on dendritic cells. *Proc. Natl. Acad. Sci. U.S.A.* **105**, 14515–14520 (2008).

56. A. Ujike, K. Takeda, A. Nakamura, S. Ebihara, K. Akiyama, T. Takai, Impaired dendritic cell maturation and increased T_H2 responses in PIR-$B^{-/-}$ mice. *Nat. Immunol.* **3**, 542–548 (2002).

57. Y. Mitsuhashi, A. Nakamura, S. Endo, K. Takeda, T. Yabe-Wada, T. Nukiwa, T. Takai, Regulation of plasmacytoid dendritic cell responses by PIR-B. *Blood* **120**, 3256–3259 (2012).

58. K. M. Dhodapkar, J. L. Kaufman, M. Ehlers, D. K. Banerjee, E. Bonvini, S. Koenig, R. M. Steinman, J. V. Ravetch, M. V. Dhodapkar, Selective blockade of inhibitory Fcγ receptor enables human dendritic cell maturation with IL-12p70 production and immunity to antibody-coated tumor cells. *Proc. Natl. Acad. Sci. U.S.A.* **102**, 2910–2915 (2005).

59. A. M. Boruchov, G. Heller, M. C. Veri, E. Bonvini, J. V. Ravetch, J. W. Young, Activating and inhibitory IgG Fc receptors on human DCs mediate opposing functions. *J. Clin. Invest.* **115**, 2914–2923 (2005).

60. A. M. Kalergis, J. V. Ravetch, Inducing tumor immunity through the selective engagement of activating Fcgamma receptors on dendritic cells. *J. Exp. Med.* **195**, 1653–1659 (2002).

61. A. L. Blasius, M. Cella, J. Maldonado, T. Takai, M. Colonna, Siglec-H is an IPC-specific receptor that modulates type I IFN secretion through DAP12. *Blood* **107**, 2474–2476 (2006).

62. H. Takagi, T. Fukaya, K. Eizumi, Y. Sato, K. Sato, A. Shibazaki, H. Otsuka, A. Hijikata, T. Watanabe, O. Ohara, T. Kaisho, B. Malissen, K. Sato, Plasmacytoid dendritic cells are crucial for the initiation of inflammation and T cell immunity in vivo. *Immunity* **35**, 958–971 (2011).

63. H. Schmitt, S. Sell, J. Koch, M. Seefried, S. Sonnewald, C. Daniel, T. H. Winkler, L. Nitschke, Siglec-H protects from virus-triggered severe systemic autoimmunity. *J. Exp. Med.* **213**, 1627–1644 (2016).

64. S. Wan, C. Xia, L. Morel, IL-6 produced by dendritic cells from lupus-prone mice inhibits $CD4^{+}CD25^{+}$ T cell regulatory functions. *J. Immunol.* **178**, 271–279 (2007).

65. A. Sang, Y. Y. Zheng, Y. Yin, I. Dozmorov, H. Li, H. C. Hsu, J. D. Mountz, L. Morel, Dysregulated cytokine production by dendritic cells modulates B cell responses in the NZM2410 mouse model of lupus. *PLOS ONE* **9**, e102151 (2014).

66. S. L. Rowland, J. M. Riggs, S. Gilfillan, M. Bugatti, W. Vermi, R. Kolbeck, E. R. Unanue, M. A. Sanjuan, M. Colonna, Early, transient depletion of plasmacytoid dendritic cells ameliorates autoimmunity in a lupus model. *J. Exp. Med.* **211**, 1977–1991 (2014).

67. G. Wu, K. Hirabayashi, S. Sato, N. Akiyama, T. Akiyama, K. Shiota, S. Yagi, DNA methylation profile of Aire-deficient mouse medullary thymic epithelial cells. *BMC Immunol.* **13**, 58 (2012).

68. T. Morinaga, S. Yanase, A. Okamoto, N. Yamaguchi, N. Yamaguchi, Recruitment of Lyn from endomembranes to the plasma membrane through calcium-dependent cell-cell interactions upon polarization of inducible Lyn-expressing MDCK cells. *Sci. Rep.* **7**, 493 (2017).

69. E. Tsantikos, T. A. Gottschalk, E. L'Estrange-Stranieri, C. A. O'Brien, A. L. Raftery, L. C. Wickramasinghe, J. L. McQualter, G. P. Anderson, M. L. Hibbs, Enhanced Lyn activity causes

severe, progressive emphysema and lung cancer. *Am. J. Respir. Cell Mol. Biol.* **69**, 99–112 (2023).

70. G. Dong, M. You, L. Ding, H. Fan, F. Liu, D. Ren, Y. Hou, STING negatively regulates double-stranded DNA-activated JAK1-STAT1 signaling via SHP-1/2 in B cells. *Mol. Cells* **38**, 441–451 (2015).

71. L. Du, B. Wang, M. Wu, W. Chen, W. Wang, W. Diao, M. Ding, W. Chen, W. Cao, H. Guo, G. Zhang, LINC00926 promotes progression of renal cell carcinoma via regulating miR-30a-5p/SOX4 axis and activating IFNγ-JAK2-STAT1 pathway. *Cancer Lett.* **578**, 216463 (2023).

72. S. Dallari, M. Macal, M. E. Loureiro, Y. Jo, L. Swanson, C. Hesser, P. Ghosh, E. I. Zuniga, Src family kinases Fyn and Lyn are constitutively activated and mediate plasmacytoid dendritic cell responses. *Nat. Commun.* **8**, 14830 (2017).

73. A. Paul, T. H. Tang, S. K. Ng, Interferon regulatory factor 9 structure and regulation. *Front. Immunol.* **9**, 1831 (2018).

74. E. Kong, Y. Li, P. Ma, Y. Zhang, R. Ding, T. Hua, M. Yang, H. Yuan, Lyn-mediated glycolysis enhancement of microglia contributes to neuropathic pain through facilitating IRF5 nuclear translocation in spinal dorsal horn. *J. Cell. Mol. Med.* **27**, 1664–1681 (2023).

75. A. Pellerin, K. Yasuda, A. Cohen-Bucay, V. Sandra, P. Shukla, B. K. Horne Jr., K. Nundel, G. A. Viglianti, Y. Xie, U. Klein, Y. Tan, R. G. Bonegio, I. R. Rifkin, Monoallelic IRF5 deficiency in B cells prevents murine lupus. *JCI Insight* **6**, e141395 (2021).

76. D. A. Savitsky, H. Yanai, T. Tamura, T. Taniguchi, K. Honda, Contribution of IRF5 in B cells to the development of murine SLE-like disease through its transcriptional control of the IgG2a locus. *Proc. Natl. Acad. Sci. U.S.A.* **107**, 10154–10159 (2010).

77. D. Feng, L. Yang, X. Bi, R. C. Stone, P. Patel, B. J. Barnes, Irf5-Deficient mice are protected from pristane-induced lupus via increased Th2 cytokines and altered IgG class switching. *Eur. J. Immunol.* **42**, 1477–1487 (2012).

78. S. De, B. Zhang, T. Shih, S. Singh, A. Winkler, R. Donnelly, B. J. Barnes, B cell-intrinsic role for IRF5 in TLR9/BCR-induced human B cell activation, proliferation, and plasmablast differentiation. *Front. Immunol.* **8**, 1938 (2017).

79. D. C. Otero, D. P. Baker, M. David, IRF7-dependent IFN-β production in response to RANKL promotes medullary thymic epithelial cell development. *J. Immunol.* **190**, 3289–3298 (2013).

80. A. J. Bonito, C. Aloman, M. I. Fiel, N. M. Danzl, S. Cha, E. G. Weinstein, S. Jeong, Y. Choi, M. C. Walsh, K. Alexandropoulos, Medullary thymic epithelial cell depletion leads to autoimmune hepatitis. *J. Clin. Invest.* **123**, 3510–3524 (2013).

81. M. Lakso, J. G. Pichel, J. R. Gorman, B. Sauer, Y. Okamoto, E. Lee, F. W. Alt, H. Westphal, Efficient in vivo manipulation of mouse genomic sequences at the zygote stage. *Proc. Natl. Acad. Sci. U.S.A.* **93**, 5860–5865 (1996).

82. Y. Xu, S. J. Beavitt, K. W. Harder, M. L. Hibbs, D. M. Tarlinton, The activation and subsequent regulatory roles of Lyn and CD19 after B cell receptor ligation are independent. *J. Immunol.* **169**, 6910–6918 (2002).

83. T. Kapanadze, J. Gamrekelashvili, S. Sablotny, F. N. Schroth, Y. Xu, R. Chen, S. Rong, N. Shushakova, F. Gueler, H. Haller, F. P. Limbourg, Validation of CSF-1 receptor (CD115) staining for analysis of murine monocytes by flow cytometry. *J. Leukoc. Biol.* **115**, 573–582 (2024).

84. B. Z. Qian, J. Li, H. Zhang, T. Kitamura, J. Zhang, L. R. Campion, E. A. Kaiser, L. A. Snyder, J. W. Pollard, CCL2 recruits inflammatory monocytes to facilitate breast-tumour metastasis. *Nature* **475**, 222–225 (2011).

85. F. L. Craciun, A. K. Ajay, D. Hoffmann, J. Saikumar, S. L. Fabian, V. Bijol, B. D. Humphreys, V. S. Vaidya, Pharmacological and genetic depletion of fibrinogen protects from kidney fibrosis. *Am. J. Physiol. Renal Physiol.* **307**, F471–484 (2014).

86. K. De Filippo, R. B. Henderson, M. Laschinger, N. Hogg, Neutrophil chemokines KC and macrophage-inflammatory protein-2 are newly synthesized by tissue macrophages using distinct TLR signaling pathways. *J. Immunol.* **180**, 4308–4315 (2008).

87. L. Overbergh, A. Giulietti, D. Valckx, R. Decallonne, R. Bouillon, C. Mathieu, The use of real-time reverse transcriptase PCR for the quantification of cytokine gene expression. *J. Biomol. Tech.* **14**, 33–43 (2003).

88. K. Kokura, Y. Kuromi, T. Endo, N. Anzai, Y. Kazuki, M. Oshimura, T. Ohbayashi, A kidney injury molecule-1 (Kim-1) gene reporter in a mouse artificial chromosome: The responsiveness to cisplatin toxicity in immortalized mouse kidney S3 cells. *J. Gene Med.* **18**, 273–281 (2016).

89. K. I. Tanaka, I. Kanazawa, J. B. Richards, D. Goltzman, T. Sugimoto, Modulators of Fam210a and roles of Fam210a in the function of myoblasts. *Calcif. Tissue Int.* **106**, 533–540 (2020).

90. K. S. Kim, H. W. Choi, H. E. Yoon, I. Y. Kim, Reactive oxygen species generated by NADPH oxidase 2 and 4 are required for chondrogenic differentiation. *J. Biol. Chem.* **285**, 40294–40302 (2010).

91. P. Lin, X. Lan, F. Chen, Y. Yang, Y. Jin, A. Wang, Reference gene selection for real-time quantitative PCR analysis of the mouse uterus in the peri-implantation period. *PLOS ONE* **8**, e62462 (2013).

92. A. Ruiz-Villalba, A. Mattiotti, Q. D. Gunst, S. Cano-Ballesteros, M. J. van den Hoff, J. M. Ruijter, Reference genes for gene expression studies in the mouse heart. *Sci. Rep.* **7**, 24 (2017).
